# *De Novo* Sequencing and Characterization of the Floral Transcriptome of *Dendrocalamus latiflorus* (Poaceae: Bambusoideae)

**DOI:** 10.1371/journal.pone.0042082

**Published:** 2012-08-14

**Authors:** Xue-Mei Zhang, Lei Zhao, Zachary Larson-Rabin, De-Zhu Li, Zhen-Hua Guo

**Affiliations:** Plant Germplasm and Genomics Center, Germplasm Bank of Wild Species, Kunming Institute of Botany, Chinese Academy of Sciences, Kunming, Yunnan, China; Yale School of Medicine, United States of America

## Abstract

**Background:**

Transcriptome sequencing can be used to determine gene sequences and transcript abundance in non-model species, and the advent of next-generation sequencing (NGS) technologies has greatly decreased the cost and time required for this process. Transcriptome data are especially desirable in bamboo species, as certain members constitute an economically and culturally important group of mostly semelparous plants with remarkable flowering features, yet little bamboo genomic research has been performed. Here we present, for the first time, extensive sequence and transcript abundance data for the floral transcriptome of a key bamboo species, *Dendrocalamus latiflorus,* obtained using the Illumina GAII sequencing platform. Our further goal was to identify patterns of gene expression during bamboo flower development.

**Results:**

Approximately 96 million sequencing reads were generated and assembled *de novo*, yielding 146,395 high quality unigenes with an average length of 461 bp. Of these, 80,418 were identified as putative homologs of annotated sequences in the public protein databases, of which 290 were associated with the floral transition and 47 were related to flower development. Digital abundance analysis identified 26,529 transcripts differentially enriched between two developmental stages, young flower buds and older developing flowers. Unigenes found at each stage were categorized according to their putative functional categories. These sequence and putative function data comprise a resource for future investigation of the floral transition and flower development in bamboo species.

**Conclusions:**

Our results present the first broad survey of a bamboo floral transcriptome. Although it will be necessary to validate the functions carried out by these genes, these results represent a starting point for future functional research on *D. latiflorus* and related species.

## Introduction

The advent of next-generation sequencing (NGS) technologies such as the Illumina Solexa, Roche 454, and ABI SOLiD platforms has revolutionized biological research by providing genomic and transcriptomic data cheaply and rapidly [1]. Advanced research in many areas, including resequencing [2], small RNA expression profiling [3], DNA methylation [4], and *de novo* transcriptome sequencing (RNA-Seq) of non-model organisms, has been performed (e.g. [5], [6], [7], [8], [9]). RNA-Seq enables high-throughput sequencing of double-stranded cDNA fragments, followed either by direct mapping of the sequences to a reference genome, or by *de novo* sequence assembly for annotation [10], [11]. The Illumina deep sequencing technology, which generates large-scale reads (75–150 bp) at lower costs, has been especially useful for *de novo* transcriptome studies [12], [13], [14], [15], [16]. This method has led to a dramatic acceleration in gene discovery [17], [18], [19] and rapidly broadened our understanding of the complexity of gene regulation and gene networks [20], [21], [22]. However, many taxonomic groups that are important for theoretical and/or practical reasons have not been sufficiently explored, and the bamboos constitute such a case.

Bamboos (Bambusoideae) belong to the monophyletic BEP clade (Bambusoideae, Ehrhartoideae, Pooideae) in grass family (Poaceae), and consist of woody and herbaceous varieties. Woody bamboos are arborescent and perennial plants characterized by their woody stems and infrequent sexual reproduction with flowering intervals ranging from several years to more than a hundred years [23], [24]. They are primarily distributed in Asia, South America and Africa, from lowlands to alpine habitats, with many playing important roles in their ecosystems like providing food or shelter for rare or endangered animals, such as the giant panda, mountain gorilla, lemur, mountain bongo, *Tylonycteris pachypus* and *Geochelone yniphora*. Woody bamboos have also been a significant natural resource with a long history of varied uses, ranging from food to raw materials for construction and manufacturing [24], [25], [26], [27]. However, these resources, and the animals and human communities that depend on them, are now threatened, since up to half of the woody bamboo species in the world are in danger of extinction [28], [29], [30], [31], [32]. The problem of wild bamboo sustainability is exacerbated by two reproductive traits common to the more economically and culturally important woody species: semelparity and mast flowering with long intermast periods [32]. Semelparity is not surprising since these species are grasses. However, the enigmatically long intermast period, from 3 to 150 years depending on the species, makes breeding programs with these “crop” plants impossible. Moreover, even if the problem of the long intermast period could be solved, pollen abortion, or sterility causing fruitlessness, would still hamper breeding efforts in bamboo [33]. Determination of the genetic pathways and specific genes involved in bamboo flowering and flower development could be beneficial for both humans and wildlife. However, limited by the availability of tissue samples or genomic information, little research has been performed to address these issues. Several putative flowering-related genes have been identified from certain bamboo species [34], [35], [36], [37], [38] and environmental and chemical manipulations have been found to induce bamboo flowering *in vitro* [39], but the genetic control of bamboo reproductive development continues to be an under-researched area.

The goal of this study was to characterize the transcriptomes of developing flowers in the bamboo species *Dendrocalamus latiflorus*, using high-throughput Illumina GAII sequencing. *D. latiflorus* is one of the most important bamboo species, because of its use in food and construction, and limited molecular research on its flowering has already been performed [34], [35], [38]. To determine the genes involved in floral development of this species, transcripts from two phases of flower growth were isolated, quantified and sequenced. These transcriptome sequences were then annotated by BLASTing against public databases. Subsequently, the annotated sequences were clustered into putative functional categories using the Gene Ontology (GO) framework and grouped into pathways using the Kyoto Encyclopedia of Genes and Genomes (KEGG). Finally, *D. latiflorus* ESTs were assigned putative homologs in model species, including *Arabidopsis thaliana*, *Oryza sativa*, *Brachypodium distachyon* and other relatives in the grass family to determine whether a hierarchy of gene regulation may persist between these species. This study represents the first exploration of the *D. latiflorus* inflorescence transcriptome using large-scale high-throughput sequencing, and the results described herein may serve to guide further gene expression and functional genomic studies in bamboos.

## Results and Discussion

### cDNA sequence generation and *de novo* assembly

A total of approximately 96 million (∼52 million and ∼44 million for phases 1 and 2, respectively) 75 bp paired-end reads were obtained after cleaning and quality checks were performed (cf. Materials and Methods). Assembly of reads resulted in 316, 821 and 293,274 contigs with mean sizes of 180 bp and 182 bp for phases 1 and 2, respectively (Table 1). Paired-end joining produced 226,593 and 208,014 scaffolds with mean sizes of 273 bp and 277 bp in phases 1 and 2, respectively. After further gap filling, the scaffolds were assembled into 109,022 unigenes for phase 1 with a mean length of 425 bp and 101,682 unigenes for phase 2 with a mean length of 429 bp (Table 1). Clustering via TGICL software [40] was used to generate 146,395 unigenes (about 67.5 Mb total length of unigenes) from phases 1 and 2 with a mean size of 461 bp (cf. Materials and Methods). The size distributions of contigs, scaffolds and unigenes were compiled (Figure S1).

**Table 1:** Statistical summary of cDNA sequences of *D. latiflorus* generated by the Illumina GAII platform.

	Phase 1	Phase 2	Total
Number of reads	52,548,610	43,966,994	-
Average read length (bp)	75	75	-
Total length of reads (bp)	3,941,145,750	3,297,524,550	-
Number of contigs	316,821	293,274	-
Average length of contigs (bp)	180	182	-
Number of scaffolds	226,593	208,014	-
Average length of scaffolds (bp)	273	277	-
Number of unigenes	109,022	101,682	146,395
Average length of unigenes (bp)	425	429	461
Total length of unigenes (bp)	46,353,147	43,621, 027	67,537,137

### Functional annotation of the flower transcriptome of *D. latiflorus*

A total of 80,418 (54.9%) *D. latiflorus* unigenes were significantly matched to known genes in the public databases (Table S1), representing putative functional identifications for more than half of the assembled sequences. Previous studies have shown that approximately 87% of Arabidopsis 454-derived ESTs could be aligned to predicted genes [41], while 72% could be similarly identified in cucumber [42] and 64% in human using the RefSeq database of well-annotated human genes [43]. As such, this study succeeded in assigning putative identification to a significant proportion of the discovered *D. latiflorus* floral transcripts given the lack of genomic information for this species. In fact, “non-BLASTable” sequences have been reported in all studied plant transcriptomes, with the proportion varying from 13 to 80%, depending on the species, the sequencing depth and the parameters of the BLAST search [8], [9], [44], [45]. Excepting the technical issues derived from sequencing, biological factors may be responsible for the large population of non-BLASTable sequences, including rapidly evolved genes (having orthologs in other species but so highly divergent that efficient recognition of orthologs is precluded), species-specific genes (present in the studied species but absent from the databases) and the persistence of non-coding fractions mainly from untranslated regions of the sampled transcripts [7].

**Figure 1:**
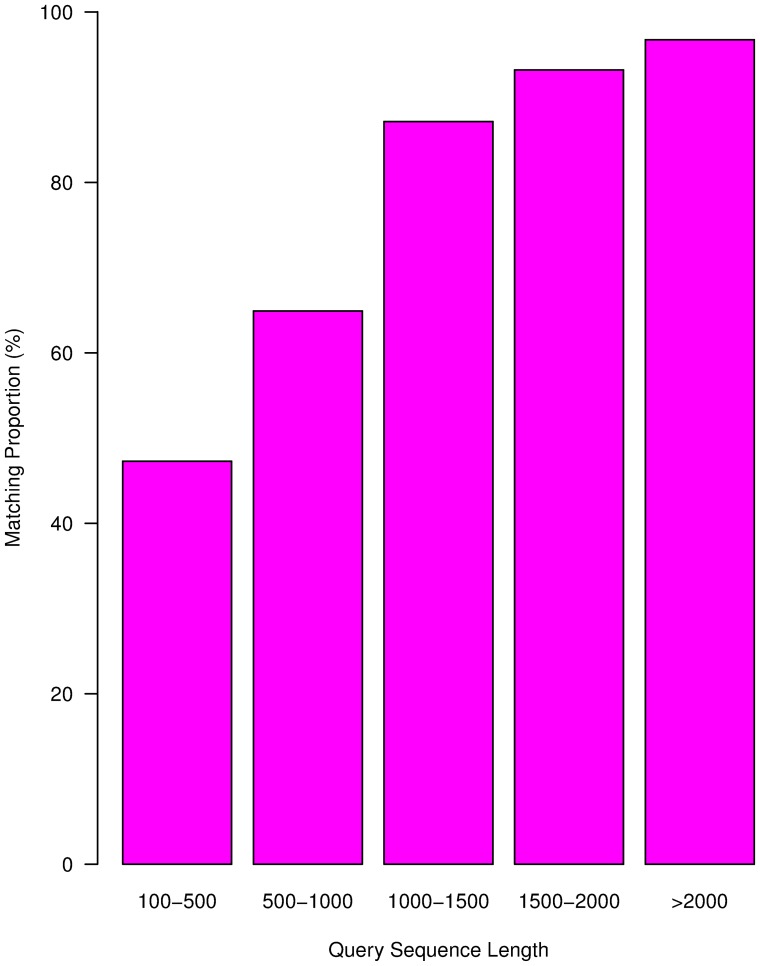
Matching percentage of *D. latiflorus* cDNA sequences with different lengths to entries in the GenBank databases.

**Figure 2:**
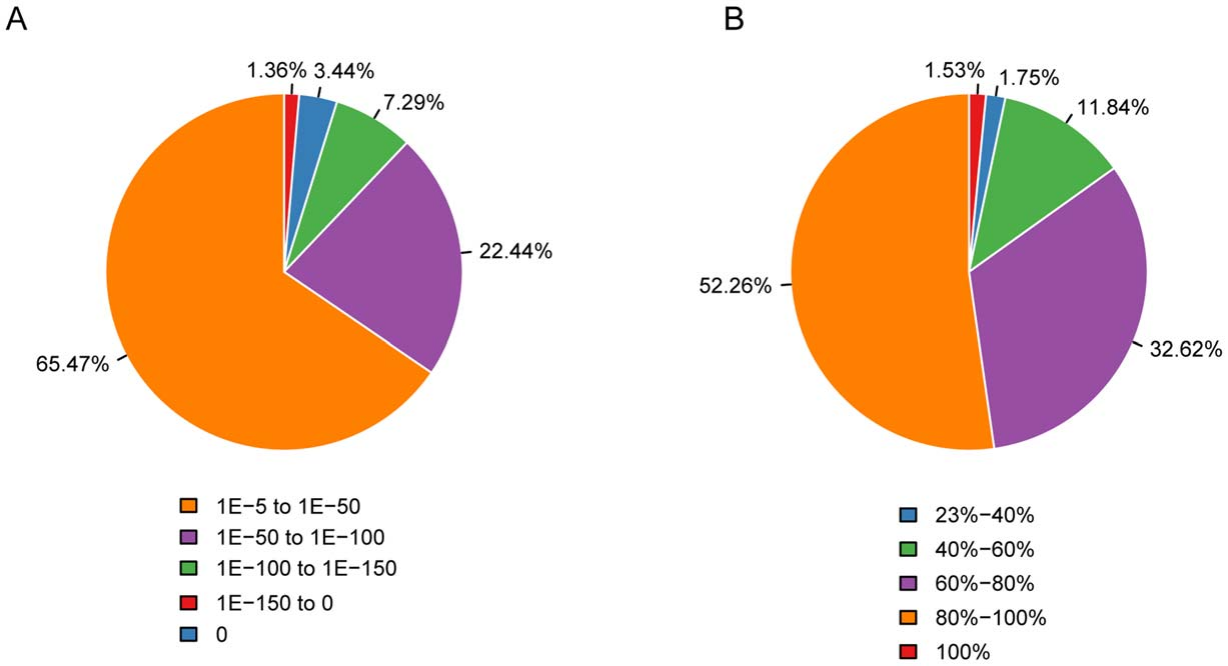
Characteristics of sequence homology of *D. latiflorus* unigenes BLASTED againsta GenBank databases. A. E-value distribution of BLAST hits for matched unigene sequences, using an E-value cutoff of 1.0E-5. B. Identity distribution of top BLAST hits for each unigene.

The assembled sequences of different lengths showed variable efficiency of matching to sequences in databases, with longer sequences showing higher match proportions (Figure 1). Match efficiency was 96.72% for sequences longer than 2,000 bp, but was 64.91% and 47.28% for sequences 500–1,000 bp and 100–500 bp in length, respectively. According to the E-value distribution of the top hits in the databases, 34.53% of the matched sequences showed strong homology (<1.0e^−50^), while 65.47% of the matched sequences showed moderate homology (between 1.0e^−5^ and 1.0e^−50^) (Figure 2A). The identity distribution pattern showed that 54.14% of the sequences had a similarity higher than 80%, while 45.86% showed similarity between 23% and 80% (Figure 2B).

**Figure 3:**
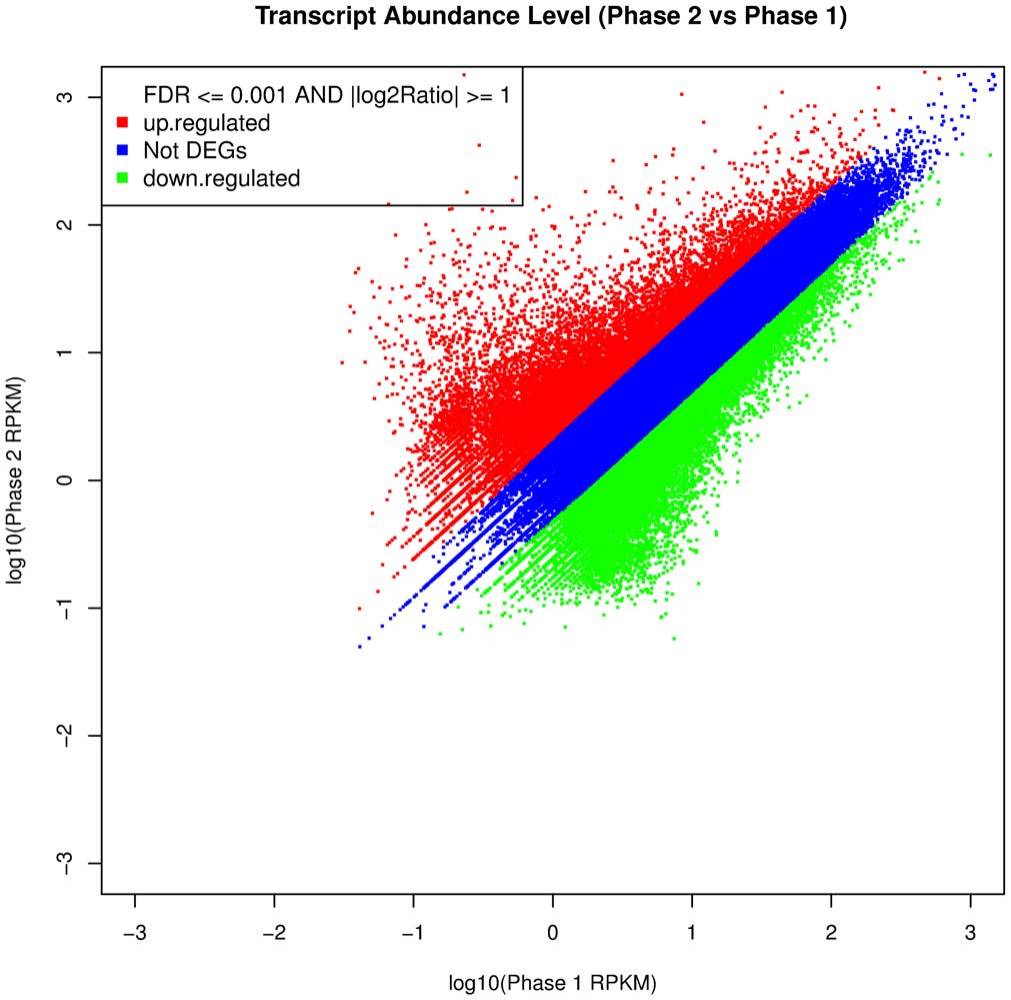
Changes of transcript abundance levels between phase 1 and phase 2.

Abundance analysis (cf. Materials and Methods) identified a total of 26,529 unigenes showing significant abundance differences between the two floral developmental phases, with 13,639 members showing increased abundance in phase 1 (4,036 of which were unique to phase 1), and 12,890 members showing increased abundance in phase 2 (3,981 of which were unique to phase 2) (Figure 3 and Table S2). The high numbers of unigenes expressed in the two phases was not unexpected given the size of the *D. latiflorus* genome, which has been regarded as a hexaploid or a complex aneuploid [46], [47], [48], [49]. This fact, combined with the lack of a complete genomic or transcriptomic sequence set for bamboo to use as a reference, increased the difficulty of unigene annotation in this study.

### Gene Ontology (GO) annotation

**Figure 4:**
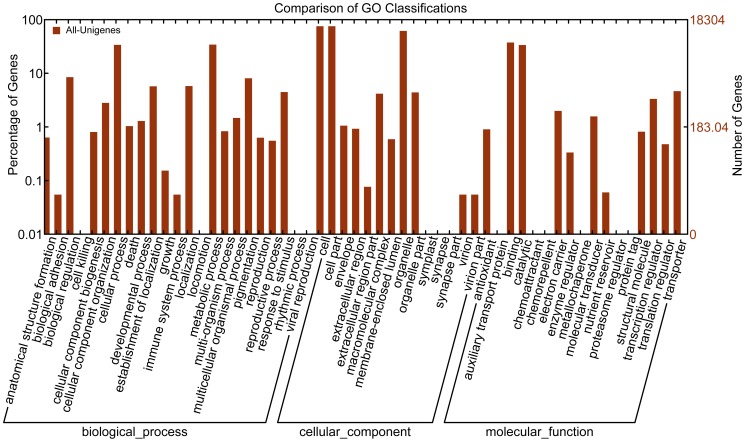
Gene Ontology classifications of assembled non-redundant unigenes.

A total of 18,304 unigenes (22.76%) were assigned to 42 functional groups using GO assignments (Figure 4), including biochemistry, growth, development, metabolism, apoptosis and immune defense. Within each of the three main categories of the GO classification scheme (biological process, cellular component and molecular function), the dominant subcategories were “cellular process”, “cell part” and “binding”, respectively. “Metabolic process”, “pigmentation”, “organelle” and “catalytic” were also well represented. However, few genes were assigned to the category “nutrient reservoir”, and no genes were found in the clusters of “cell killing”, “rhythmic process”, “viral reproduction”, “symplast”, “synapse”, “synapse part”, “auxiliary transport protein”, “chemoattractant”, “chemorepellent”, “metallochaperone”, “protein regulation” or “protein tag”.

A further functional classification of all unigenes was performed using a set of plant-specific GO slims (Figure S2). “Cytoplasm”, “plastid”, “membrane” and “mitochondrion” were the most highly represented groups, followed closely by “cellular process”, “biosynthetic process” and “metabolic process”. Genes involved in flower development (47), pollination (16), and pollen-pistil interaction (16), stress response (470), signal transduction (429), cell differentiation (43), and regulatory and epigenetic genes (25) were also represented.

In both phases of floral development, plant-specific GO Slim analysis (cf. Materials and Methods) revealed that most of the sequences were related to cellular component organization, cell cycle, nucleic acid binding, nucleic acid metabolic processes, transcription, cellular macromolecule biosynthetic processes, cellular macromolecule metabolic processes, generation of precursor metabolites and energy, and macromolecule modification. Unigenes more abundant in the phase 1 transcriptome, however, were involved in transcription activity, translation, small molecule metabolic processes and vigorous organelle development, while the phase 2 transcriptome skewed toward cellular processes, signal transduction, signal transmission, cell wall structuring, nucleic acid and chromatin binding, stress response, macromolecule metabolic processes, and cell death (Table S3). These trends were consistent with overall developmental activities during those time periods. For example, genes related to transcription, translation and general metabolism have been found to be abundant during early flower development. Conversely, transcripts involved in signaling, cell wall metabolism and cellular processes were overrepresented in flowers of late development stage [50], [51], [52], [53].

### COG annotation

**Figure 5:**
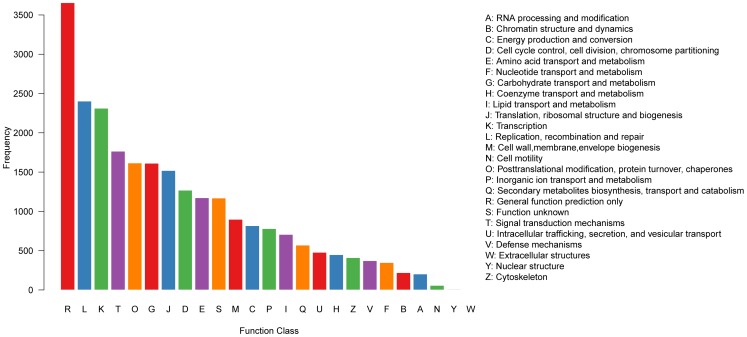
COG functional classifications of the *D. latiflorus* flower transcriptome.

Out of 80,418 hits in the public databases, 18,535 sequences were classified into 25 COG categories (Figure 5), among which “General function prediction only” represented the largest group (3,655, 14.7%), followed by “Replication, recombination and repair” (2,402, 9.68%), “Transcription” (2,312, 9.32%) and “Signal transduction mechanisms” (1,765, 7.11%). “Cell motility” (58, 0.23%), “Nuclear structure” (9, 0.04%) and “Extracellular structures” (4, 0.02%) were the smallest groups.

### KEGG pathway mapping

Referencing the 30,043 *D. latiflorus* unigenes through the KEGG database (cf. Materials and Methods) predicted a total of 265 pathways, representing compound biosynthesis, degradation, utilization and metabolism. Transcripts identified as related to the following cellular processes or components were the most abundant: chromosome (3,055 unigenes), spliceosome (2,391), ubiquitin system (2,072), DNA repair and recombination proteins (1,709), DNA replication proteins (1,262), protein kinases (1,082), purine metabolism (1,028), chaperones and folding catalysts (981), peptidases (942), pyrimidine metabolism (904) and starch and sucrose metabolism (903) (Figure S3A). The largest category was metabolism (17,326) which included carbohydrate metabolism (4,039), amino acid metabolism (2,665), biosynthesis of secondary metabolites (1,947), nucleotide metabolism (1,932), lipid metabolism (1,834), energy metabolism (1,417) and other subcategories (Figure S3B). In the secondary metabolism category, 21 subcategories comprised 1,947 unigenes, the most represented of which were phenylpropanoid biosynthesis (528), limonene and pinene degradation (283), stilbenoid, diarylheptanoid and gingerol biosynthesis (241), terpenoid backbone biosynthesis (148), flavonoid biosynthesis (133) and carotenoid biosynthesis (115), and anthocyanin biosynthesis (19) was also classified (Figure S3C).

In addition to metabolism pathways, genetic information processing genes (6,850) and environmental information processing genes (2,528) were highly represented categories. Transcription, translation, replication and repair, folding, sorting and degradation, signal transduction, membrane transport and signaling interaction were included in these categories. Separating the unigenes by transcript abundance, phase 1 enriched transcripts skewed toward the categories such as chromosome, nucleotide metabolism, DNA replication, and DNA repair and recombination proteins. Genes showing increased abundance in phase 2 were skewed toward phenylpropanoid biosynthesis, starch and sucrose metabolism, flavonoid biosynthesis, pentose and glucuronate interconversions, phenylalanine metabolism, tryptophan metabolism, fatty acid metabolism, flavone and flavonol biosynthesis, carotenoid biosynthesis, oxidative phosphorylation and apoptosis (Table S4).

### Putative *D. latiflorus* floral development transcription factors

Transcriptional regulation is mediated through the interplay between transcription factor proteins and specific cis-regulatory regions of the genome to activate gene expression and hence to bring about development or other changes [54]. As such, some transcription factors may be considered master regulators, since their actions begin a branching series of downstream effects, including the activation of other transcription factors, which ultimately lead to broad changes in the organism. This study focused on these key regulatory genes.

A total of 81 putative transcription factor families were identified in developing *D. latiflorus* flowers, with 55 and 68 showing increased unigene transcript abundance in phase 1 and phase 2, respectively (Tables S1 and S2). Many of the same unigenes were found in both samples. Putative homologs of growth-regulating factor (GRF) genes, however, were restricted to phase 1. In *Arabidopsis thaliana* and *Oryza sativa*, *GRF* genes form a small transcription factor family with 9 and 12 members, respectively [55], [56]. The GRF proteins bear two highly conserved regions, the QLQ (Gln, Leu, Gln) domain and the WRC (Trp, Arg, Cys) domain [55], [57]. Biochemical and genetic data have suggested that GRFs act as transcription activators and are part of a complex involved in regulating the morphogenesis of leaves and petals [58], [59]. The *GRF* transcripts were highly abundant in actively growing and developing tissues, such as shoot tips, flower buds and immature leaves, but less abundant in mature tissues and organs [55], [56], suggesting a role in regulating cell proliferation [56], [59]. Given the significantly high levels of *GRF* transcripts early in *D. latiflorus* flower development, these genes may operate similarly in this bamboo species.

The SBP-box genes were firstly isolated from *Antirrhinum majus* (*SBP1* and *SBP2* [60]). Members of the SBP-box gene family regulate diverse aspects of plant development. *SBP1* and *SBP2* interact with the promoter element of the floral meristem identity gene *SQUAMOSA* of *A. majus* and thus are involved in flower development [60]. The maize SBP-box gene *LG1* (*Liguleless1*) functions in the development of leaf ligules and auricules [61] and *tga1* (*teosinte glume architecture*) controls the development of its naked grains [62]. The Arabidopsis *SPL3* gene (*SQUA* promoter-binding protein-like 3, a *SBP1* ortholog) has shown activity primarily in the vegetative apical meristem, leaf primordia, inflorescence apical meristems, floral meristems and floral organ primordia. The SPL3 protein was found to bind with a conserved sequence in the promoter of floral meristem identity gene *AP1*, promoting the floral transition, and it may also be involved in inflorescence development since *35S::SPL3se* transgenic Arabidopsis produced bracts subtending flowers [63]. Also in Arabidopsis, *SPL14* and *SPL8* showed involvement in leaf development [64] and pollen sac development [65], respectively, while the microRNA-regulated *SPL9* and *SPL15* appear to control shoot maturation [66]. In tomato, the *LeSPL-CNR* (*Colorless nonripening*) gene likely regulates fruit ripening [67]. *SPL* genes may also participate in stress responses [68]. The present study found that putative homologs of *SPL2, SPL3, SPL6, SPL8, SPL9, SPL12, SPL13, SPL14,* and *SPL15* were expressed in developing *D. latiflorus* flowers, suggesting similar roles from *SPL* genes during *D. latiflorus* flower development.

The basic helix-loop-helix family (bHLH) contains genes regulating various processes of flower development, such as controlling the development of carpel margins, as well as the morphogenesis of sepals, petals, stamens and anthers in Arabidopsis and *Eschscholzia californica* [69], [70]. In the present study, 96 unigenes with bHLH-like sequences showed significantly higher abundance in phase 1, while 68 were higher in phase 2. Such prevalence of bHLH-containing transcripts early in floral development suggests that this family is as involved in flower development in *D. latiflorus* as it is in other species.

Auxin-response factors (ARFs) have been shown to regulate auxin responsive genes [71], [72]. In Arabidopsis, loss-of-function mutations in *ETTIN (ARF3)* led to aberrant perianth organ numbers and spacing, as well as regional differentiation defects for the stamens and gynoecium, indicating an involvement in regional identity determination [73]. The 24 putative *ARF* homologs from *D. latiflorus* were significantly more abundant in the young flower buds than in the older floral tissues.

The basic leucine zipper (bZIP) transcription factors regulate diverse biological processes in plants such as flower development (e.g. *PERIANTHIA* [74]), light and stress signaling (e.g. *AREB1* and *AREB2* [75]) and seed maturation (e.g. *ABI5* [76]). In developing *D. latiflorus* flowers, both phases showed similar numbers of unigenes with bZIP sequence similarity (40 and 37 for phase 1 and phase 2, respectively).

The zinc finger homeodomain (zf-HD) genes encode a group of transcriptional regulators showing high activity during Arabidopsis floral development and to a lesser extent in vegetative development [77]. Some zf-HD members were shown to be expressed in a floral-specific manner (e.g. *ATHB33* and *ATHB28*), with some members being more highly expressed in younger flowers (e.g. *ATHB21, ATHB25,* and *ATHB31*) and others more highly expressed in older flowers (e.g. *ATHB22, ATHB27* and *ATHB29*) [77]. In *D. latiflorus*, 24 and 3 putative zf-HD genes were highly abundant in young flowers and older flowers, respectively. Unigenes similar to CCAAT box binding factors (e.g. *HAP/NF-Y* complex components) were also significantly enriched in young flowers (phase 1) relative to older flowers (phase 2) (18 vs. 1). Some of the CCAAT genes have been shown to be important in regulating flowering and stress responses in plants [78], [79].

The large NAC transcription factor family was significantly represented among phase 2 transcripts, with 103 unigenes highly abundant compared with 41 unigenes abundant in phase 1. Members of this family have been implicated in diverse biological processes in other plant species, including floral and vegetative development [80], [81], [82], [83], [84], [85], auxin signaling [85], responses to abscisic acid [86], defense [87], biotic and abiotic stress [88], [89], [90], [91], [92], light responses [73], [93], [94], [95], programmed cell death [95], [96] and senescence [97], [98], [99]. It has been suggested that NAC proteins enable crosstalk between different pathways [100].

Putative homologs of WRKY transcription factors showed a similar overrepresentation, with 97 unigenes showing high abundance in phase 2 compared with 41 highly abundant unigenes in phase 1. WRKY transcription factors have been shown to be associated with senescence [101], [102] and stress responses [103]. Members of this family have been identified as important downstream components of MAPK signaling pathways that confer to resistance to both bacterial and fungal pathogens [104] They may be induced by signaling hormones such as salicylic acid [105], [106], jasmonic acid [107] and gibberellic acid [108]. The *AP2-EREBP* family was also enriched with 57 putative homologs highly abundant in phase 2 and 43 in phase 1. Members of this family perform various activities including floral organ identity specification and leaf epidermal cell patterning [109]. They can be induced by hormones such as jasmonic acid, salicylic acid and ethylene, along with other signals involved in pathogen attack, wounding and abiotic stresses, and may influence other stress and disease resistance pathways [110], [111].

The MYB transcription factors contain DNA binding domains and some have been identified as floral developmental regulators [112], [113]. *R2R3-MYB* genes have been reported to regulate various metabolic pathways, including those for phenylpropanoid metabolism [114] and tryptophan biosynthesis [115]. In Arabidopsis, *MYB21*, *MYB24* and *MYB57* are *DELLA*-repressible GA-response genes that mediate stamen filament growth [116] and *AtPAP1 (PRODUCTION OF ANTHOCYANIN PIGMENT1)* can strongly enhance the ectopic expression of flavonoid biosynthesis genes in most organs to produce intense purple pigmentation [114]. In this study, these metabolic and biological processes were significantly represented among transcripts in phase 2 (Table S4), suggesting increased MYB activity in this later stage.

Several other transcription factor families were also found. MADS-box genes have been intensely studied in model plants and many MADS family members have been shown to orchestrate floral organ specification and development [117], [118], [119]. Loss-of-function mutants of MADS-box genes have caused changes in organ identity. In the present study, 120 and 81 putative MADS genes were highly abundant in phase 2 and phase 1, respectively. A majority showed the greatest similarity to *Vitis vinifera* genes while others were homologous to rice, maize or sorghum sequences. Further investigation of *D. latiflorus* putative homologs of maize, sorghum or rice genes (e.g. *OsMADS58* [120], [121]) should provide interesting clues to flower development in the grasses.

### Detection of putative sequences related to flowering time control and flower development

The unusually infrequent nature of bamboo flowering has attracted the curiosity of scientists and laypeople alike over the centuries [23], and the biological basis of this trait, especially whether the same genes involved in flowering in other species function in bamboo species, has remained an open question. By comparing the *D. latiflorus* unigenes found in this study with the NCBI and Uniprot databases, at least 290 unigenes were discovered showing homology to known flowering-related genes from other plants (Table S5). These unigenes were compared to flowering genes from rice and temperate grasses, as well as from Arabidopsis (Figure S4).

For the photoperiod pathway, *D. latiflorus* unigenes showing homology to components of the circadian clock included *LATE ELONGATED HYPOCOTYL* (*LHY*), *PSEUDO-RESPONSE REGULATOR 1* (*PRR95*), *EARLY FLOWERING 3* (*ELF3*), *EARLY FLOWERING 4* (*ELF4*) and *CONSTITUTIVE PHOTOMORPHOGENIC 1* (*COP1*) [122]. Several homologs of *CONSTANS* (*CO*), the key regulator of photoperiod response, were also identified (*CO5, CO6, CO7* and *CO8* [123]). *CIRCADIAN CLOCK ASSOCIATED 1* (*CCA1*), *TIMING OF CAB1* (*TOC1*) and *GIGANTEA* (*GI*) [122] homologs were absent. However, a set of unigenes did show homology to the *indeterminate 1* gene (*id1*). *Id1* was first identified in maize (*Zea mays*) [124], [125] and no clear orthologs were found in Arabidopsis, whereas its ortholog in rice (known as *RID1*, *OsId1,* or *EARLY HEADING DATE 2, Ehd2*) was discovered to be a key photoperiod-independent flowering regulator [126], [127], [128].

Putative autonomous flowering time pathway genes included unigenes showing homology to *FLOWERING LOCUS D* (*FLD*), *FY*, *FCA* [123] and *DICER-LIKE 3A* (*DCL3A*) [129]. In the vernalization pathway, *D. latiflorus* putative homologs of *MULTICOPY SUPPRESSOR OF IRA1* (*MSI1*), and *VERNALIZATION INDEPENDENT INSENSITIVE 3* (*VIN3*) [123] were expressed. Each of these homologs, except *MSI1*, downregulates the flowering repressor, *FLOWERING LOCUS C* (*FLC*), and promotes the transition from vegetative to reproductive growth [129], [130], [131], [132]. Putative homologs of GA-signaling pathway genes were also found, including the gibberellin response modulators *dwarf 8* (*d8*) [133], [134] and *GAMYB* [135], and gibberellin receptor *GID1L2* [136].

Putative homologs of floral meristem identity genes like *AP1* [137], *MADS14* [138] and *MADS58* [139] were also identified, as well as other non-classified flowering-time genes (Table S5). This study did not find any unigenes with obvious similarity to *FT*, a master regulator of the transition to flowering in Arabidopsis (with apparent paralogous counterparts in rice called *RFT* and *Hd3a*); however, since the tissues used in this study were floral buds, it remains possible that a *D. latiflorus FT* homolog is involved in the transition to flowering upstream of bud emergence but is absent from flowers.

Certain flowering-time genes controlling the transition to flowering have also shown involvement in organ development, and a host of these were found to be expressed in developing *D. latiflorus* flowers in this study. For example, *CONSTANS* (*CO*) gene, a key regulator of the photoperiodic flowering response in Arabidopsis, expresses not only in shoot apical meristems and leaves, but also in inflorescences and roots [140]; *GhCO*, a homolog of *CO* gene in *Gossypium hirsutum*, expresses mainly in flower buds and mature flowers, and in ovules to a lesser extent [141]. The autonomous pathway gene *FCA* expresses at the shoot apex but also in mature leaves, inflorescences and roots [122]. Transcripts of the MYB genes have been found in young flower buds, mature flowers and fruits [69], while expression of *AP1* was found in the early development of individual flowers [140]. *RFL*, the rice *LFY* homolog, not only facilitates the transition to flowering, but also regulates the development of panicles and tillers [142]. Many MADS genes have been shown to function in floral organ specification and development [117], [118], [119], and *EMF2*, an epigenetic regulator of homeotic flower development genes including MADS genes, showed wide expression in shoots, leaves, roots, stems and inflorescences [38]. Thus, the *D. latiflorus* putative flowering-time gene homologs identified in this study may likewise have dual roles in flowering time and flower development. Forty-seven other putative flower development genes were also found, including those involved in morphogenesis of floral structures (meristem, primordia, whorl, organ number, spikelet, panicle and nectary) and development of the reproductive system (pollen and ovules) in other species (Figure S2 and Table S6).

## Conclusions

Previous studies have demonstrated the usefulness of next generation short-read DNA sequencing technology in generating genomic data for non-model organisms, and subsequently comparing the resulting sequences to model-species reference genomes [6], [143]. Despite the fascinating mystery of bamboo flowering and the economic importance of several bamboo species, little genetic or genomic research has been performed. The study presented here begins to address this shortcoming by using the Illumina GAII platform to investigate the sequences and transcript abundance levels of genes expressed in developing flowers of *D. latiflorus*. In total, 146,395 unigenes were isolated, 80,418 of which could be identified as putative homologs of annotated sequences in the public databases, with 290 being putative homologs of known flowering genes in other species. These sequences provide a starting point for the further investigation of bamboo flowering, and the 26,529 other unigenes from diverse pathways that were differentially expressed between phase 1 and phase 2 should inform research into later stages of floral development. The results provided here represent the largest genetic resource for *D. latiflorus* to date, and they could serve as the foundation for further genomics research on this species or its relatives.

## Materials and Methods

### Sample preparation and RNA isolation

**Figure 6:**
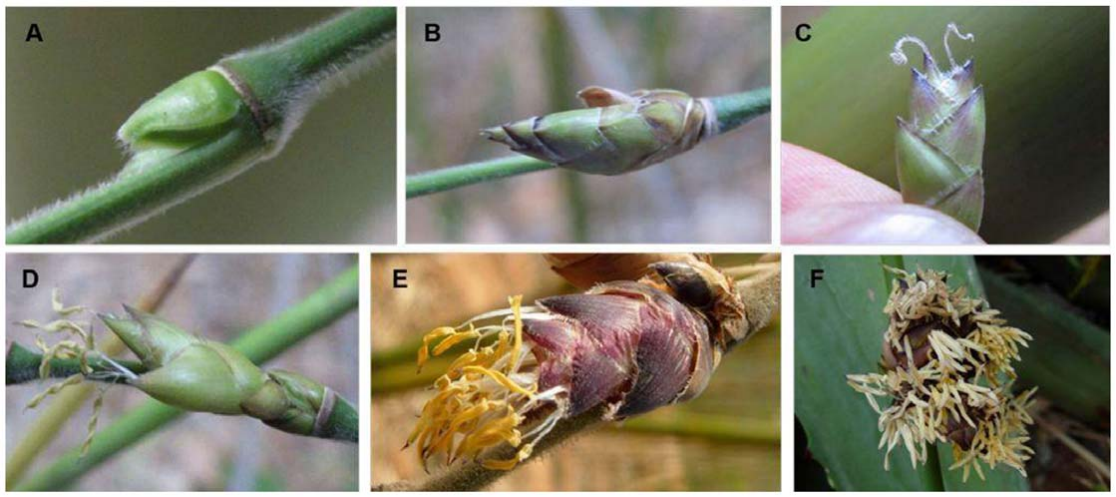
Examples of sampled floral tissues. A. Representatives of flowers collected to produce the sample for phase 1 (flower buds ≤5 mm). B–F. Representatives of flowers collected to produce the sample for phase 2 (flower buds ≥5 mm). B. Flowers with pistils and stamens not yet emerging from glumes. C. Flowers from which only pistils have emerged from glumes. D. Flowers with both pistils and stamens emerging from glumes. E. Flowers for which anthers have already dehisced. F. Senescing flowers.

*D. latiflorus* flowers were collected from each of the 14 ramets of one flowering genet between 12:05 pm and 12:30 pm on April 14, 2009 near Xiaozhai village, Pengpu town, Mile county of Yunnan Province in southwest China. All collected flowers were grouped into two sizes based on bud length. The phase 1 sample consisted of buds ≤5 mm and the phase 2 sample consisted of buds ≥5 mm (see Figure 6 for details). Samples were immediately frozen in liquid nitrogen and stored at −80°C until RNA was extracted. Total RNA of each sample was isolated using RNAiso Plus (TaKaRa). RNA quality was characterized initially on an agarose gel and NanoDrop ND1000 spectrophotometer (NanoDrop Technologies, Wilmington, DE, USA) and then further assessed by RIN (RNA Integrity Number) value (>8.0) using an Agilent 2100 Bioanalyzer (Santa Clara, CA, USA).

### Library preparation and sequencing

The cDNA libraries were prepared according to the manufacturer's instructions (Illumina, San Diego, CA). Poly-A mRNA molecules were purified using Sera-mag Magnetic Oligo (dT) Beads (Illumina) from 20 µg total RNA from each sample and eluted with 10 mM Tris–HCl. To avoid priming bias during cDNA synthesis, the purified mRNA was first fragmented into small pieces using RNA Fragmentation Reagents (Ambion, Austin, TX, USA) before cDNA synthesis. The cleaved mRNA fragments were converted to double-stranded cDNA using random hexamer primers (Illumina) with the SuperScript Double-Stranded cDNA Synthesis kit (Invitrogen, Camarillo, CA). The resulting cDNAs were purified using the QiaQuick PCR Purification Kit (Qiagen, Valencia, CA) and then subjected to end-repair and phosphorylation using T4 DNA polymerase, Klenow DNA polymerase and T4 PNK (NEB, Ipswich, MA, USA). Repaired cDNA fragments were 3′ adenylated using Klenow Exo- (Illumina), producing cDNA fragments with a single ‘A’ base overhang at their 3′ ends for subsequent adapter ligation. Illumina paired-end adapters were ligated to the ends of these 3′ adenylated cDNA fragments. To select a size range of templates for downstream enrichment, the products of the ligation reaction were purified on a 2% TAE-agarose gel (Certified Low-Range Ultra Agarose, BioRad, Hercules, CA). A range of cDNA fragments (200±25 bp) was excised from the gel and extracted using QIAquick Gel Extraction Kit (Qiagen). Fifteen rounds of PCR amplification were performed to enrich the purified cDNA template using primers complementary to the ends of the adapters [PCR Primer PE 1.0 and PCR Primer PE 2.0 (Illumina) with Phusion DNA Polymerase. Finally, after validating on an Agilent Technologies 2100 Bioanalyzer using the Agilent DNA 1000 chip kit, the cDNA library products were sequenced on a paired-end flow cell using an Illumina Genome Analyzer II at Beijing Genomics Institute (BGI) in Shenzhen, China.

### Data processing and *de novo* assembly

Because the algorithms used in *de novo* transcriptome construction of the short reads provided by the Illumina platform may be severely inhibited by sequencing errors, a stringent cDNA sequence filtering process was employed to select clean reads. First, Illumina's Failed-Chastity filter software was used to remove raw reads that fell into the relation “failed-chastity ≤1”, with a chastity threshold of 0.6 on the first 25 cycles. Second, all raw reads showing signs of adaptor contamination or ambiguous trace peaks (denoted with an “N” in the sequence trace) were removed. Finally, raw reads showing more than 10% of bases with a Phred-scaled probability (Q) less than 20 were discarded.

The resulting clean short reads that showed sufficient overlap with other reads were joined using the SOAPdenovo software [144] to generate longer, contiguous sequences (i.e., contigs). Contigs were rejected unless their K-mers were conjoined along an unambiguous path. The identity of the different contigs from a transcript, and their distance, were recognized by mapping clean reads back to the corresponding contigs based on their paired end information. Joining of these contigs and filling of the unknown interspaces (i.e., gaps) using “Ns” (i.e., ambiguous base calls) resulted in the generation of scaffolds. Finally, the gaps of scaffolds were filled using the paired-end clean reads according to their sequence complementarity to scaffolds, resulting in sequences with the fewest Ns that also could not be further extended on either end. Such sequences were defined as unigenes. To obtain distinct sequences, the unigenes from the two different phases were clustered using the TGI Clustering tool [40].

Unigenes were then aligned to a series of protein databases using BLASTx (E-value <10^−5^). Databases included the NCBI non-redundant protein (Nr), Swiss-Prot, the Kyoto Encyclopedia of Genes and Genomes (KEGG) pathway [145] and the Cluster of Orthologous Groups of proteins (COG) (http://www.ncbi.nlm.nih.gov/COG/) [146] databases. Sequence directionality was assigned according to the best alignments. When the different databases gave different results, the following priority structure was used to choose one unigene: NCBI Nr, Swiss-Prot, KEGG and COG. When a unigene failed to align to any of the four databases, ESTScan [147] was used to predict its coding regions and ascertain its sequence direction.

### Unigene transcript abundance analysis

To analyze unigene transcript abundance levels, the uniquely mapped reads for a specific unigene were counted by mapping reads of each phase to *de novo* assembled distinct sequences using SOAP2 software [148], and the RPKM (Reads Per Kb per Million reads) values were computed as proposed by Mortazavi et al. [10]. Unigene transcript abundance differences between the two floral development phases of *D. latiflorus* were obtained from RPKM values using a method modified from Audic's proposal [149]. Fold changes for each unigene between sample pairs (Phase 2 vs. Phase 1) were computed as the ratio of the RPKM values. If the value of Phase2 RPKM or Phase1 RPKM was 0, 0.001 was used instead of 0 to measure the fold change. The significance of differential transcript abundance was computed using the FDR (False Discovery Rate) control method [150] to justify the p-value, and only unigenes with an absolute fold change ≥2 and a FDR significance score ≤0.001 were used for subsequent steps of the analysis. The formula to determine the significant p-value between two samples was defined as follows.
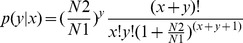
In the formula, N1 and N2 represent the total number of clean reads mapped to All-unigenes in each sample, and x and y represent respectively the number of clean reads mapped to a common unigene in phase 1 and phase 2.

### Gene annotation

To assign putative gene function, unigenes were searched against the NCBI Nr and Swiss-Prot databases using local BLASTx with an E-value cutoff of 10^−5^. Estimates of the numbers of annotated unigenes that matched to genes from the two databases were made and the unigene lists were then filtered to remove duplications. Functional categories of the predicted genes were obtained by applying gene ontology (GO) terms (http://www.geneontology.org) [151] to the Nr database annotation using the Blast2GO program [152], and summarized using WEGO software [153]. Then the GO annotations of the unigenes were mapped to the plant-specific GO slim ontology using the map2slim script (www.geneontology.org/GO.slims.shtml) [154] (p-value <0.05), and final classification of the unigenes was based on these GO slims. To evaluate the completeness of our transcriptome library and the effectiveness of our annotation process, we searched the annotated unigene sequences for the possible functions involved in COG classifications. To summarize which pathways are active in *D. latiflorus* flowers, we mapped the annotated sequences to the reference canonical pathways in KEGG. To identify transcription factor families represented in our samples, unigene sequences were searched against the complete list of transcription factor protein sequences of the Plant Transcription Factor Database (PlnTFDB: http://plntfdb.bio.uni-potsdam.de/v3.0/downloads.php) [155] using BLASTX with an E-value cutoff of ≤10^−5^.

To assign putative biological functions and pathway involvement to the unigenes, enrichment analysis was carried out (Table S3, Table S4). First, all unigenes showing significant transcript abundance differences between phases (“differentially abundant unigenes”) were mapped to the GO and KEGG pathway databases, and then the numbers of unigenes for every GO term and KO term were calculated, respectively. To compare these unigenes to the whole *D. latiflorus* transcriptome background, the hypergeometric test was applied to find significantly enriched GO and KO terms from the set of differentially abundant unigenes. The formula for the gene enrichment test was
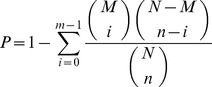
in which N represents the total number of unigenes with GO and KEGG pathway annotation; n represents the number of differentially abundant unigenes in N; M represents the number of unigenes that were annotated to certain GO or KO terms; and m represents the number of differentially abundant unigenes in M. The initially obtained p-values were then adjusted using a Bonferroni Correction and a corrected p-value of 0.05 was adopted as a threshold.

## Supporting Information

Figure S1 **Overview of** ***D. latiflorus*** **flower transcriptome sequencing and assembly.** A. Length distribution of contigs from phase 1; B. Length distribution of contigs from phase 2; C. Length distribution of scaffolds from phase 1; D. Length distribution of scaffolds from phase 2; E. Length distribution of unigenes from phase 1; F. Length distribution of unigenes from phase 2; G. Length distribution of all unigenes.
(PDF)

Figure S2 **Plant-specific GO Slim terms for the** ***D. latiflorus*** **florally expressed unigenes.** The bar chart provides the plant-specific GO slim terms enriched for unigenes expressed in *D. latiflorus* flowers.
(PDF)

Figure S3 **KEGG pathway categories assigned with** ***D. latiflorus*** **flower unigenes.** A. Top KEGG pathways highly represented by *D. latiflorus* flower unigenes. B. KEGG metabolism pathways. C. KEGG secondary metabolism pathways.
(PDF)

Figure S4 **Pathways regulating the floral transition in Arabidopsis, rice and temperate grasses (Compiled from references** [**123]**, [129], [156], [157], [158], [159], [160], [161], [162], [163], [164], [165], [166], [167], [168], [169], [170], [171], [172], [173], [174], [175], [**176**] **) and putative homologous unigenes in** ***D. latiflorus.*** **.** I. Pathways in Arabidopsis. II. Pathways in rice. III. Pathways in temperate grasses. Arrows indicate a promotive effect; broken arrows indicate a possible relationship; perpendicular lines indicate a repressive effect. Genes are shown in rectangles and proteins are shown in circles. Vernalization pathway genes are shown in blue, autonomous pathway genes in pink, photoperiod pathway genes in green, and GA-signaling pathway genes in purple. Floral pathway integrators are shown in red, and floral meristem identity genes are shown in grey. Labels: v indicates vernalization; ld indicates long days; sd indicates short days. Bold and italicized typeface indicates genes and proteins for which similar unigenes were found in *D. latiflorus* in the present study. * indicates the gene was first identified in maize but lacks homologs in Arabidopsis.
(PDF)

Table S1 **Top BLAST hits from public databases.** A list of the top results from BLASTING *D. latiflorus* unigenes against public databases (E-value cutoff of 10^−5^).
(XLS)

Table S2 **List of unigenes showing differential transcript abundance.** A list of the unigenes showing differential transcript abundance in flowers of phase 1 and phase 2.
(XLS)

Table S3 **Enriched plant-specific GO terms for unigenes showing differential transcript abundance.** A list of the plant-specific GO terms enriched for unigenes showing higher transcript abundance in phase 1 and phase 2, respectively (p<0.05).
(PDF)

Table S4 **Enriched KEGG pathways for unigenes showing differential transcript abundance.** A list of KEGG pathways enriched for unigenes showing higher transcript abundance in phase 1 and phase 2, respectively (p<0.05).
(PDF)

Table S5 **Representatives of putative flowering-time genes in** ***D.latiflorus*** **.** A list of the *D. latiflorus* putative flowering time control genes and their possible functions.
(PDF)

Table S6 **Putative flower development genes in** ***D. latiflorus*** **.** A list of the genes putatively related to flower development in *D. latiflorus* and their possible functions.
(XLS)
