# Supplementary material for: De Novo Sequencing and Characterization of the Floral Transcriptome of Dendrocalamus latiflorus (Poaceae: Bambusoideae)
Source: PLoS One. 2012 Aug 14;7(8):e42082. doi: 10.1371/journal.pone.0042082 (PMC3419236; doi:10.1371/journal.pone.0042082)
Supplement: Figure S1 — Overview of D. latiflorus flower transcriptome sequencing and assembly. A. Length distribution of contigs from phase 1; B. Length distribution of contigs from phase 2; C. Length distribution of scaffolds from phase 1; D. Length distribution of scaffolds from phase 2; E. Length distribution of unigenes from phase 1; F. Length distribution of unigenes from phase 2; G. Length distribution of all unigenes. (PDF) [file pone.0042082.s001.pdf]

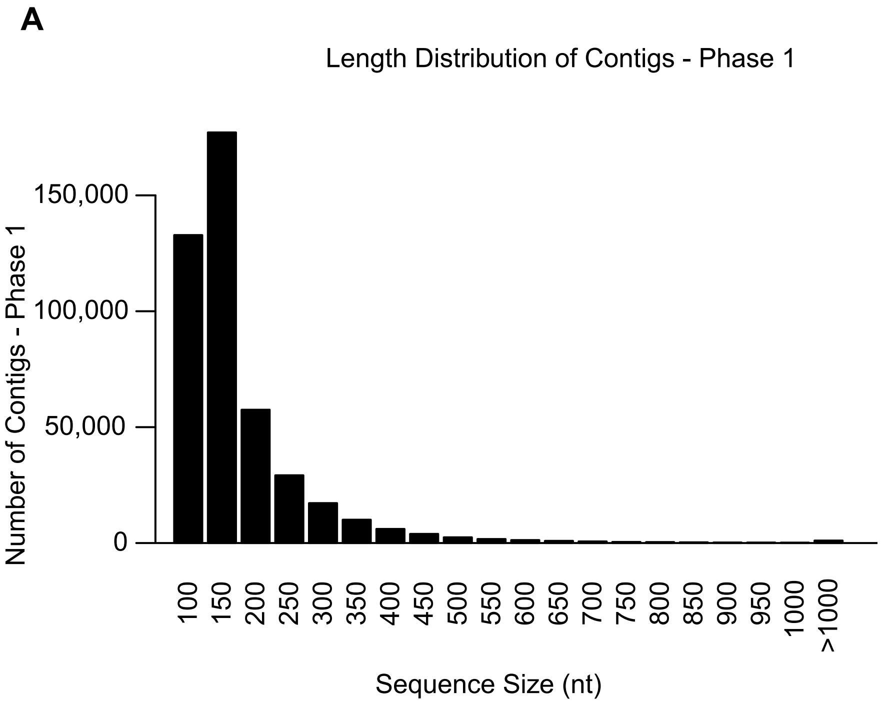
A
Length Distribution of Contigs - Phase 1
Number of Contigs - Phase 1
150,000
100,000
50,000
0
100
150
200
250
300
350
400
450
500
550
600
650
700
750
800
850
900
950
1000
>1000
Sequence Size (nt)

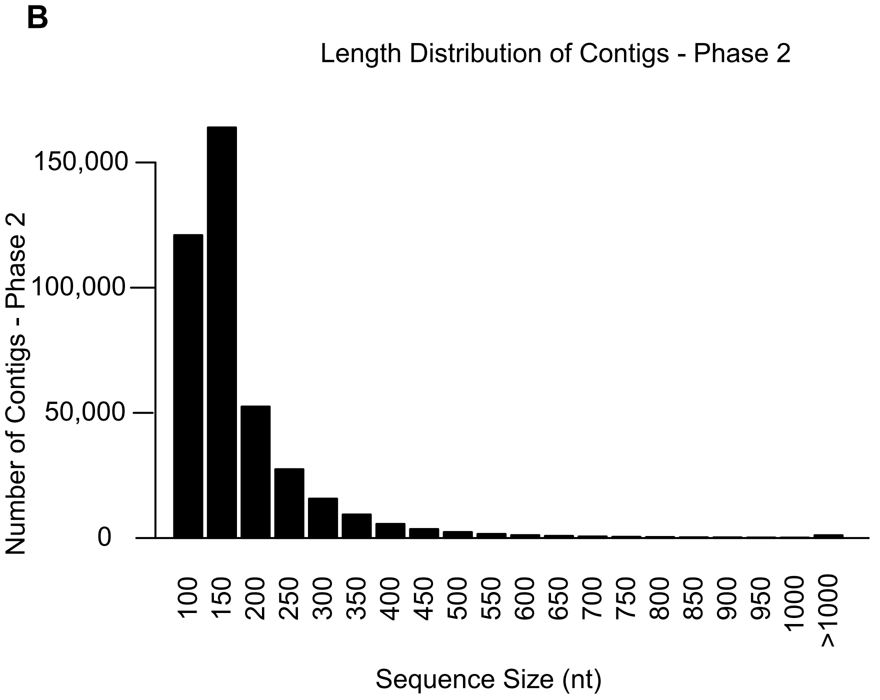
B
Length Distribution of Contigs - Phase 2
Number of Contigs - Phase 2
150,000
100,000
50,000
0
100
150
200
250
300
350
400
450
500
550
600
650
700
750
800
850
900
950
1000
>1000
Sequence Size (nt)

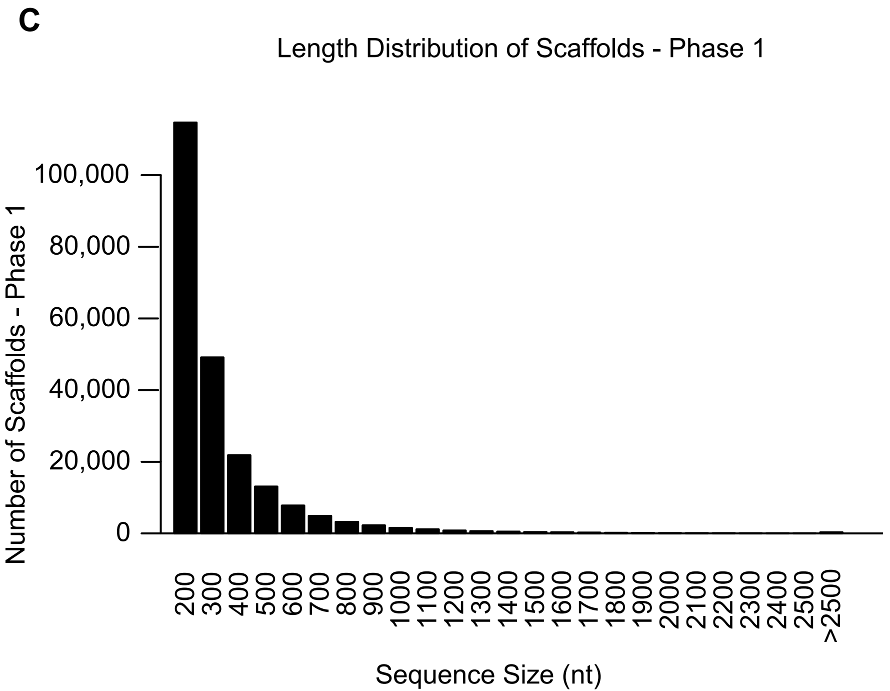
C
Length Distribution of Scaffolds - Phase 1
Number of Scaffolds - Phase 1
100,000
80,000
60,000
40,000
20,000
0
200
300
400
500
600
700
800
900
1000
1100
1200
1300
1400
1500
1600
1700
1800
1900
2000
2100
2200
2300
2400
2500
>2500
Sequence Size (nt)

D

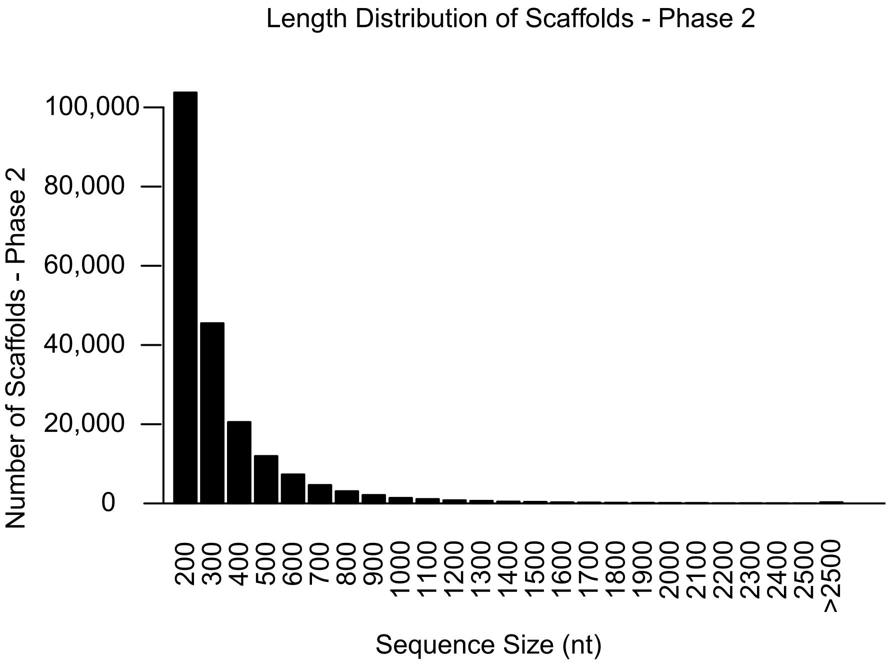

Length Distribution of Scaffolds - Phase 2
Number of Scaffolds - Phase 2
100,000
80,000
60,000
40,000
20,000
0
200
300
400
500
600
700
800
900
1000
1100
1200
1300
1400
1500
1600
1700
1800
1900
2000
2100
2200
2300
2400
2500
>2500
Sequence Size (nt)

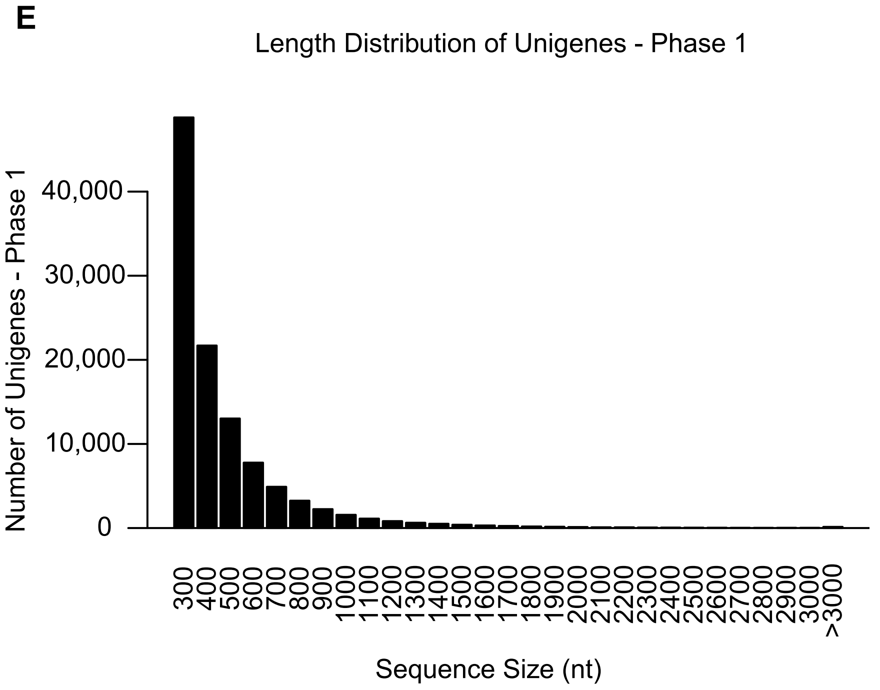
E
Length Distribution of Unigenes - Phase 1
Number of Unigenes - Phase 1
40,000
30,000
20,000
10,000
0
300
400
500
600
700
800
900
1000
1100
1200
1300
1400
1500
1600
1700
1800
1900
2000
2100
2200
2300
2400
2500
2600
2700
2800
2900
3000
>3000
Sequence Size (nt)

F

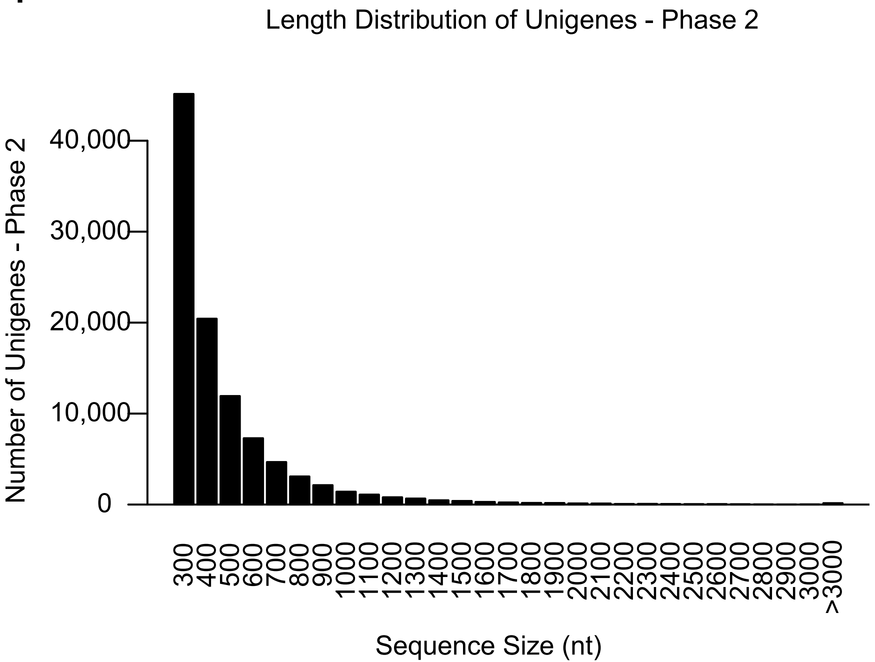
Length Distribution of Unigenes - Phase 2
Number of Unigenes - Phase 2
40,000
30,000
20,000
10,000
0
300
400
500
600
700
800
900
1000
1100
1200
1300
1400
1500
1600
1700
1800
1900
2000
2100
2200
2300
2400
2500
2600
2700
2800
2900
3000
>3000
Sequence Size (nt)

G

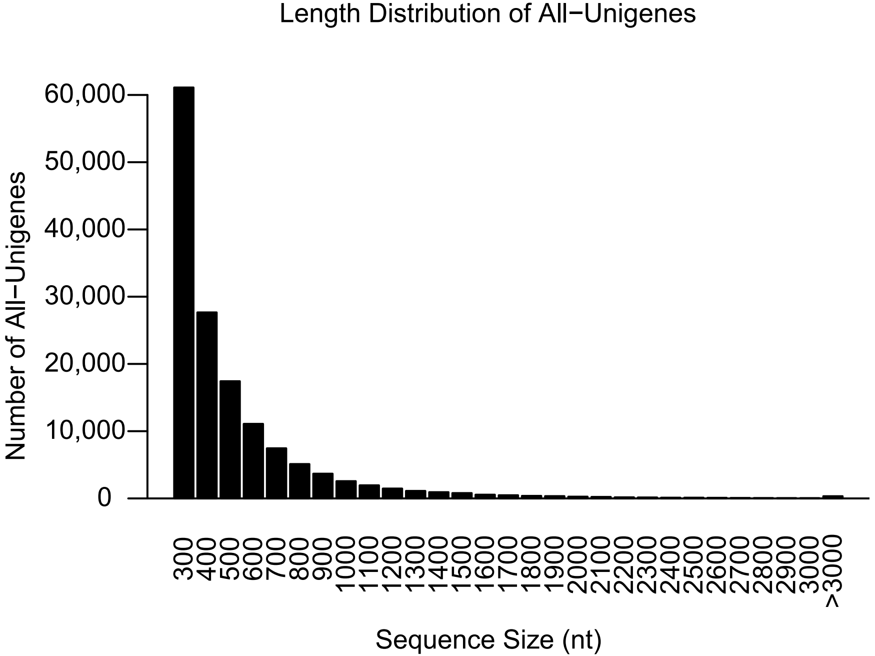

Length Distribution of All−Unigenes
60,000
50,000
40,000
30,000
20,000
10,000
0
Number of All−Unigenes
300
400
500
600
700
800
900
1000
1100
1200
1300
1400
1500
1600
1700
1800
1900
2000
2100
2200
2300
2400
2500
2600
2700
2800
2900
3000
>3000
Sequence Size (nt)
