# Supplementary figures and images for: De Novo Sequencing and Characterization of the Floral Transcriptome of Dendrocalamus latiflorus (Poaceae: Bambusoideae)
Source: PLoS One. 2012 Aug 14;7(8):e42082. doi: 10.1371/journal.pone.0042082 (PMC3419236; doi:10.1371/journal.pone.0042082)

# Number of Unigenes

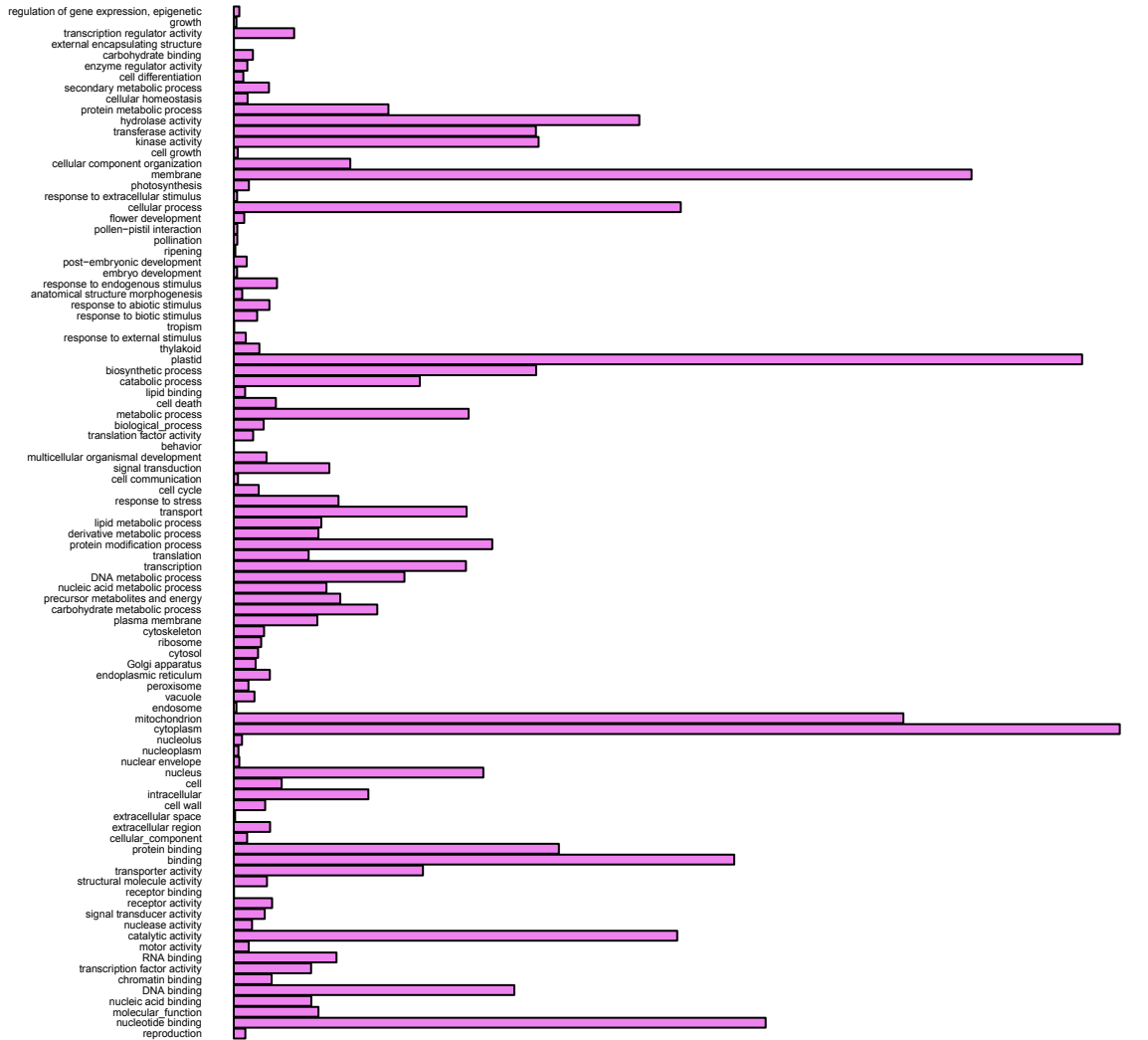

Supplement: Figure S2 — Plant-specific GO Slim terms for the D. latiflorus florally expressed unigenes. The bar chart provides the plant-specific GO slim terms enriched for unigenes expressed in D. latiflorus flowers. (PDF) [file pone.0042082.s002.pdf]

**A**

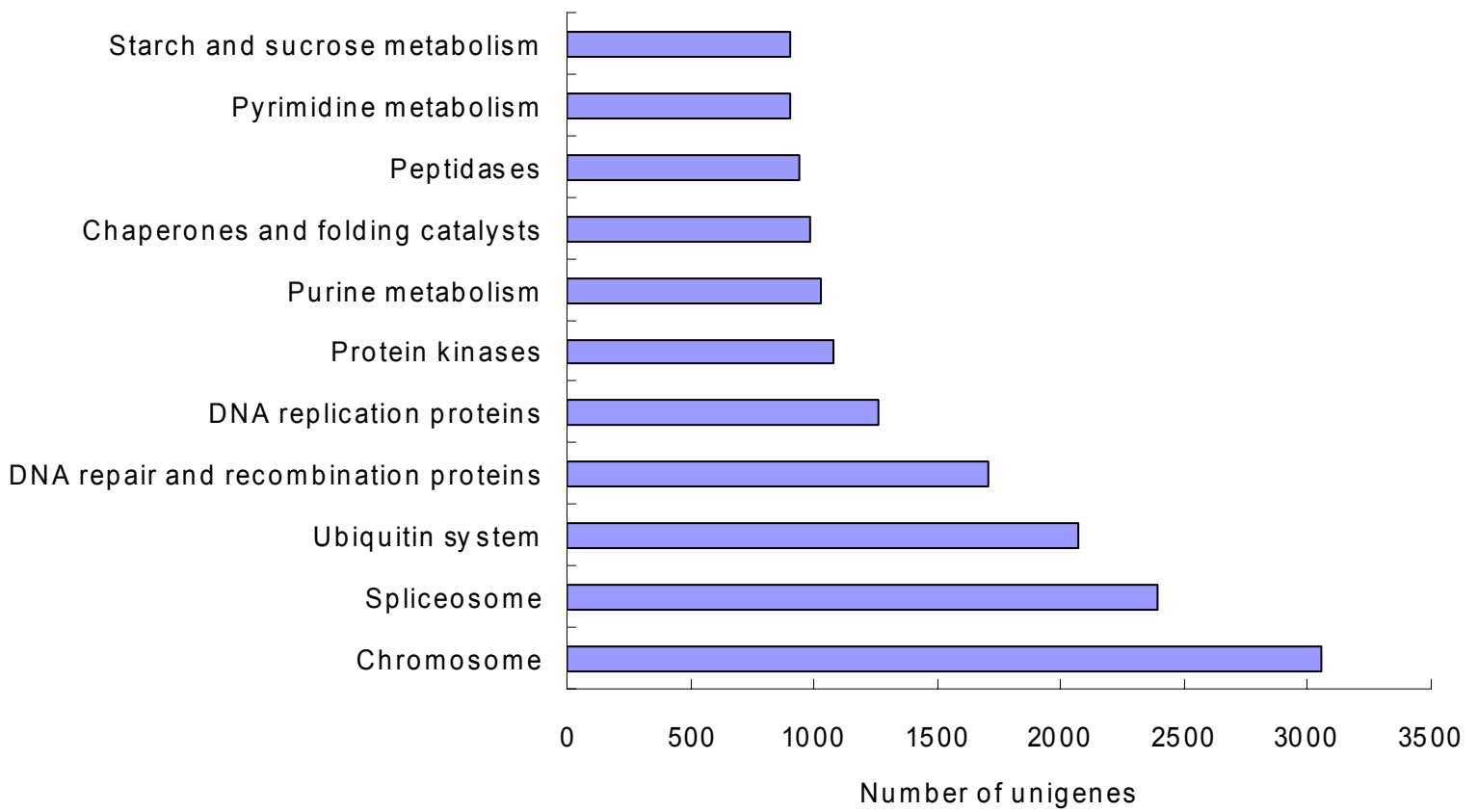

**B**

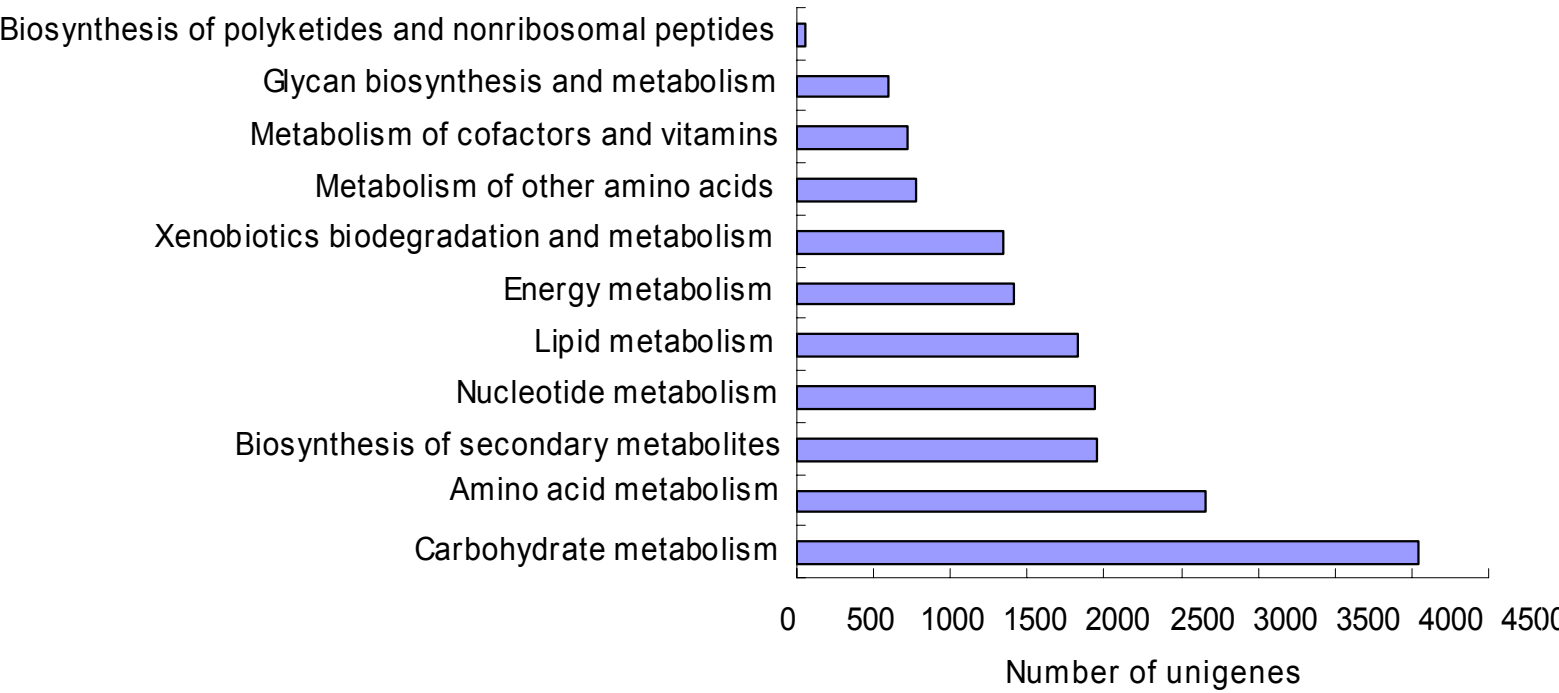

C

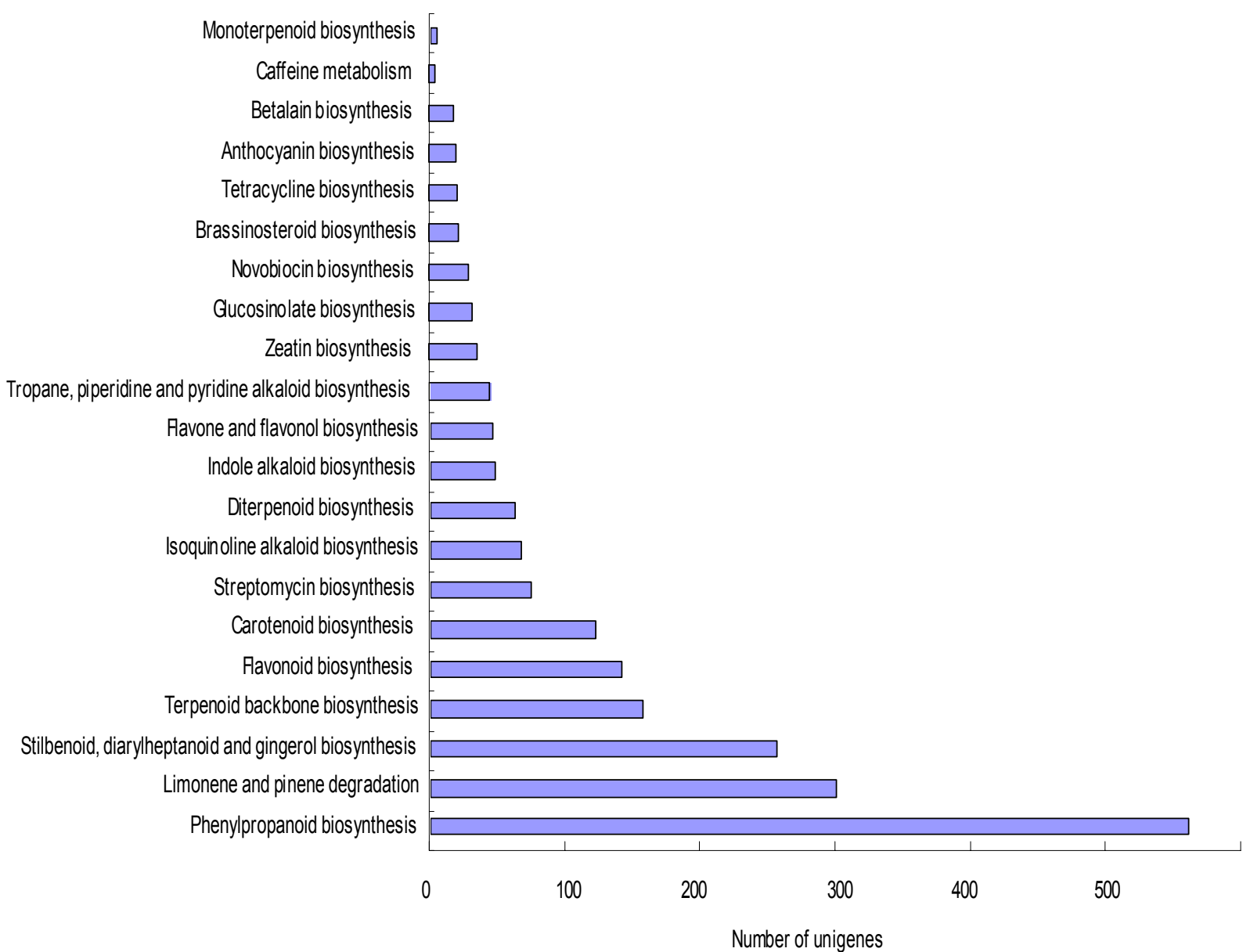

Supplement: Figure S3 — KEGG pathway categories assigned with D. latiflorus flower unigenes. A. Top KEGG pathways highly represented by D. latiflorus flower unigenes. B. KEGG metabolism pathways. C. KEGG secondary metabolism pathways. (PDF) [file pone.0042082.s003.pdf]
