# Supplementary material for: De Novo Sequencing and Characterization of the Floral Transcriptome of Dendrocalamus latiflorus (Poaceae: Bambusoideae)
Source: PLoS One. 2012 Aug 14;7(8):e42082. doi: 10.1371/journal.pone.0042082 (PMC3419236; doi:10.1371/journal.pone.0042082)

I. Arabidopsis

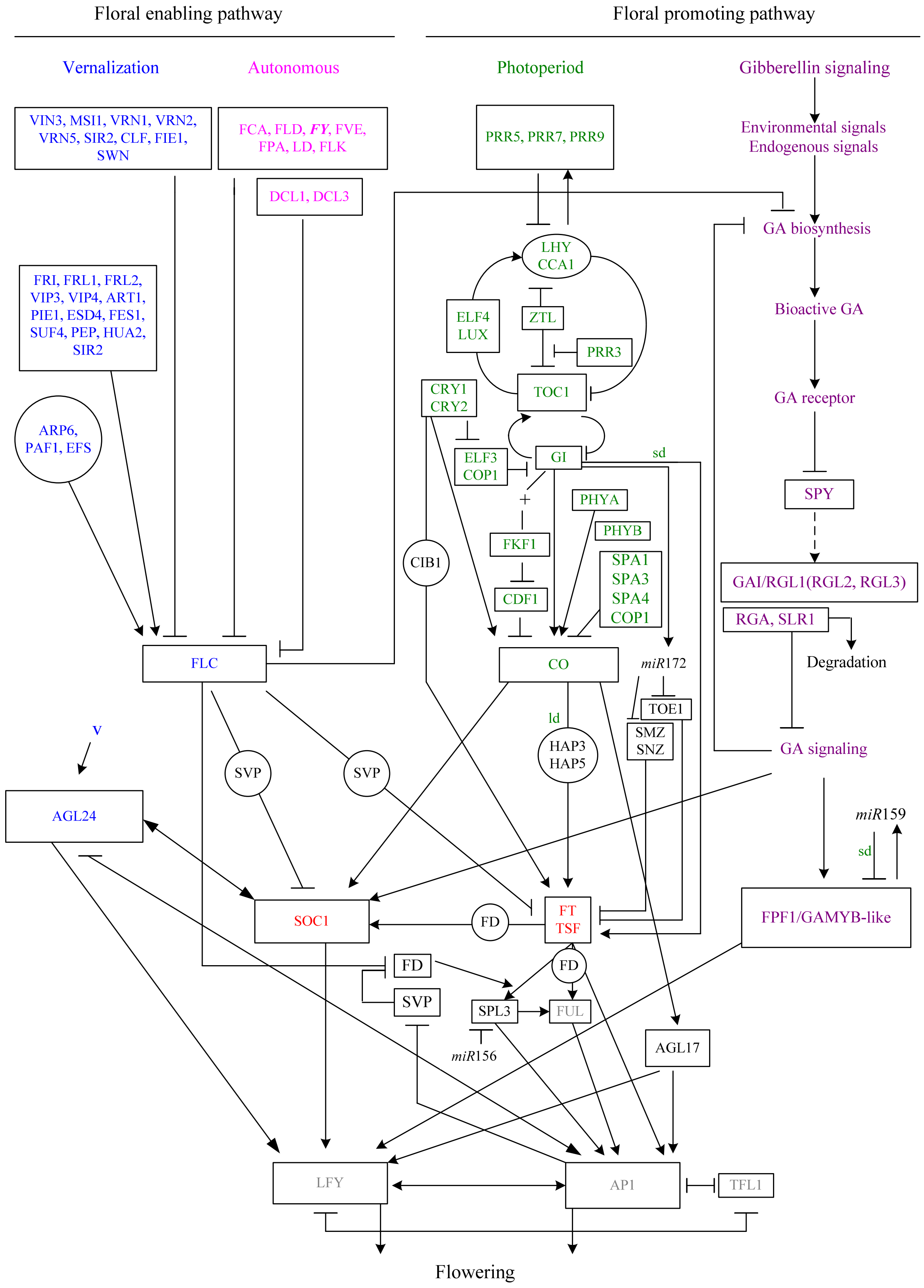

II. Rice

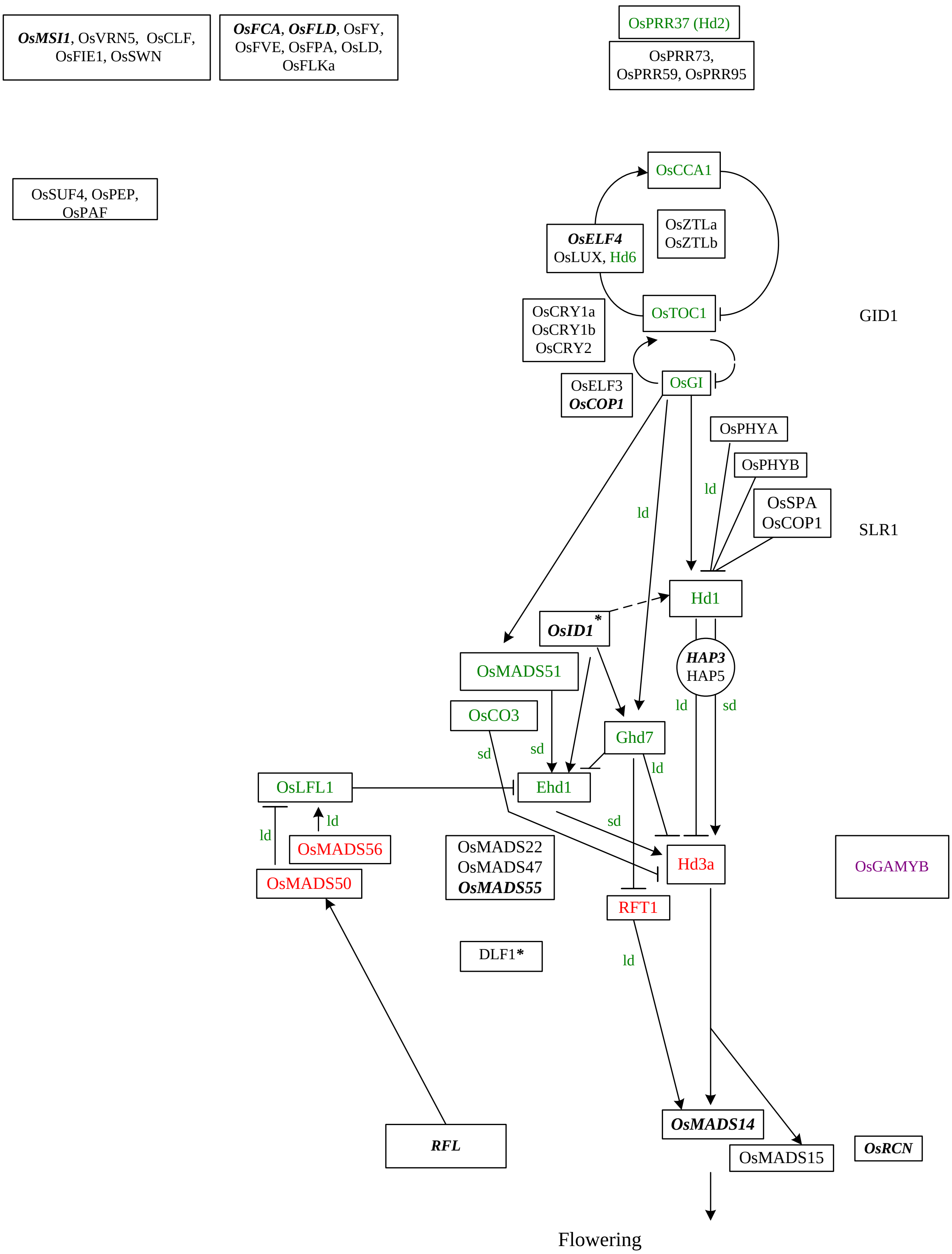

OsMSI1, OsVRN5, OsCLF, OsFIE1, OsSWN
OsFCA, OsFLD, OsFY, OsFVE, OsFPA, OsLD, OsFLKa
OsPRR37 (Hd2)
OsPRR73, OsPRR59, OsPRR95
OsSUF4, OsPEP, OsPAF
OsCCA1
OsZTLa OsZTLb
OsELF4 OsLUX, Hd6
OsTOC1
GID1
OsCRY1a OsCRY1b OsCRY2
OsGI
OsELF3 OsCOP1
OsPHYA
OsPHYB
OsSPA OsCOP1
SLR1
ld
Hd1
OsID1*
OsMADS51
HAP3 HAP5
OsCO3
Ghd7
sd
Ehd1
OsLFL1
OsMADS56
OsMADS50
OsMADS22 OsMADS47 OsMADS55
Hd3a
RFT1
OsGAMYB
DLF1*
RFL
OsMADS14
OsMADS15
OsRCN
Flowering

III. Temperate grasses

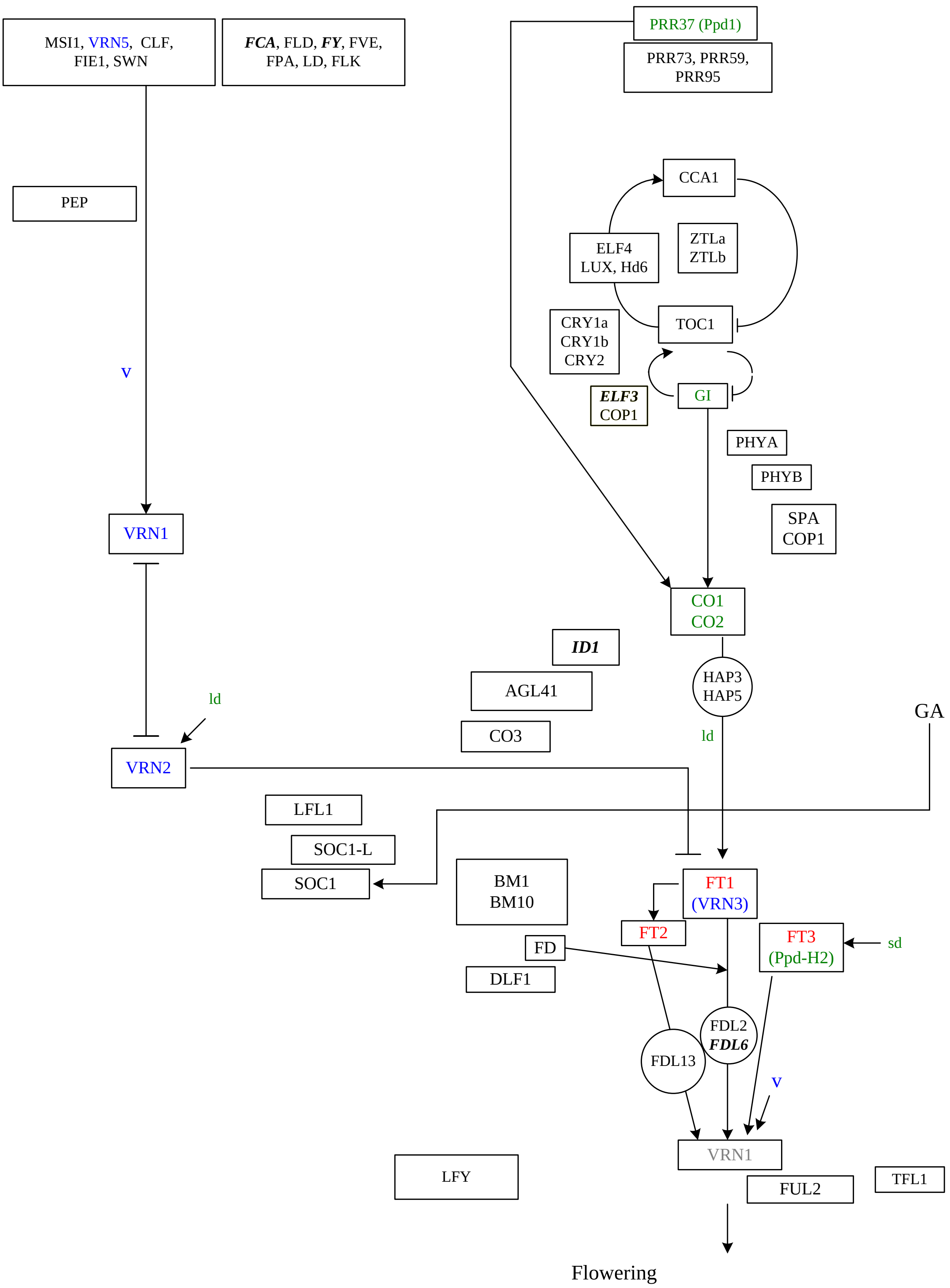

Supplement: Figure S4 — Pathways regulating the floral transition in Arabidopsis, rice and temperate grasses (Compiled from references [123] , [129], [156], [157], [158], [159], [160], [161], [162], [163], [164], [165], [166], [167], [168], [169], [170], [171], [172], [173], [174], [175], [176] ) and putative homologous unigenes in D. latiflorus. . I. Pathways in Arabidopsis. II. Pathways in rice. III. Pathways in temperate grasses. Arrows indicate a promotive effect; broken arrows indicate a possible relationship; perpendicular lines indicate a repressive effect. Genes are shown in rectangles and proteins are shown in circles. Vernalization pathway genes are shown in blue, autonomous pathway genes in pink, photoperiod pathway genes in green, and GA-signaling pathway genes in purple. Floral pathway integrators are shown in red, and floral meristem identity genes are shown in grey. Labels: v indicates vernalization; ld indicates long days; sd indicates short days. Bold and italicized typeface indicates genes and proteins for which similar unigenes were found in D. latiflorus in the present study. * indicates the gene was first identified in maize but lacks homologs in Arabidopsis. (PDF) [file pone.0042082.s004.pdf]
