# Supplementary material for: De Novo Sequencing and Characterization of the Floral Transcriptome of Dendrocalamus latiflorus (Poaceae: Bambusoideae)
Source: PLoS One. 2012 Aug 14;7(8):e42082. doi: 10.1371/journal.pone.0042082 (PMC3419236; doi:10.1371/journal.pone.0042082)
Supplement: Table S3 — Enriched plant-specific GO terms for unigenes showing differential transcript abundance. A list of the plant-specific GO terms enriched for unigenes showing higher transcript abundance in phase 1 and phase 2, respectively (p<0.05). (PDF) [file pone.0042082.s007.pdf]

Plant-specific GO terms significantly enriched for unigenes showing higher transcription in phase 1 (P<0.05)

GO term ID	Name	adjusted P value	Unigene number
GO:0051234	establishment of localization	3.91E-10	38
GO:0006810	transport	3.91E-10	38
GO:0005215	transporter activity	7.02E-09	29
GO:0005634	nucleus	9.80E-09	172
GO:0090304	nucleic acid metabolic process	4.58E-08	215
GO:0007049	cell cycle	4.65E-08	33
GO:0030528	transcription regulator activity	5.21E-07	90
GO:0006350	transcription	5.23E-07	140
GO:0016020	membrane	8.17E-07	238
GO:0044260	cellular macromolecule metabolic process	8.81E-07	351
GO:0044444	cytoplasmic part	2.01E-06	521

GO:0003824	catalytic activity	5.13E-05	469
GO:0005783	endoplasmic reticulum	8.55E-05	3
GO:0006091	generation of precursor metabolites and energy	1.06E-04	20
GO:0003677	DNA binding	1.25E-04	165
GO:0043170	macromolecule metabolic process	2.52E-04	347
GO:0016043	cellular component organization	3.64E-04	70
GO:0005737	cytoplasm	3.85E-04	403
GO:0005623	cell	6.75E-04	32
GO:0006629	lipid metabolic process	6.95E-04	17
GO:0006412	translation	6.95E-04	15
GO:0003700	transcription factor activity	8.57E-04	51
GO:0006519	cellular amino acid and derivative metabolic process	9.72E-04	19
GO:0044281	small molecule metabolic process	9.72E-04	19

GO:0005773	vacuole	9.72E-04	1
GO:0034641	cellular nitrogen compound metabolic process	0.001229	225
GO:0006139	nucleobase, nucleoside, nucleotide and nucleic acid metabolic process	0.001229	225
GO:0006807	nitrogen compound metabolic process	0.001229	225
GO:0016301	kinase activity	0.001393	165
GO:0016772	transferase activity, transferring phosphorus-containing groups	0.001393	165
GO:0034645	cellular macromolecule biosynthetic process	0.001996	151
GO:0009059	macromolecule biosynthetic process	0.001996	151
GO:0044249	cellular biosynthetic process	0.001996	151
GO:0003676	nucleic acid binding	0.002572	214
GO:0005975	carbohydrate metabolic process	0.003366	39
GO:0044446	intracellular organelle part	0.003366	1
GO:0044422	organelle part	0.003366	1

GO:0044428	nuclear part	0.003366	1
GO:0005198	structural molecule activity	0.003366	5
GO:0044424	intracellular part	0.00363	1050
GO:0008152	metabolic process	0.003814	491
GO:0009056	catabolic process	0.003814	44
GO:0005739	mitochondrion	0.005142	224
GO:0005829	cytosol	0.005302	3
GO:0015979	photosynthesis	0.008717	1
GO:0005794	Golgi apparatus	0.013616	3
GO:0004872	receptor activity	0.014886	27
GO:0030246	carbohydrate binding	0.018145	16
GO:0005840	ribosome	0.018791	5
GO:0032991	macromolecular complex	0.018791	5

GO:0030529	ribonucleoprotein complex	0.018791	5
GO:0006259	DNA metabolic process	0.021505	13
GO:0005576	extracellular region	0.022077	26
GO:0005730	nucleolus	0.025346	3
GO:0043412	macromolecule modification	0.029804	131
GO:0006464	protein modification process	0.029804	131
GO:0004518	nuclease activity	0.038541	2
GO:0016788	hydrolase activity, acting on ester bonds	0.038541	2
GO:0044464	cell part	0.042332	13
GO:0016462	pyrophosphatase activity	0.042332	13
GO:0017111	nucleoside-triphosphatase activity	0.042332	13
GO:0003774	motor activity	0.042332	13
GO:0016817	hydrolase activity, acting on acid anhydrides	0.042332	13

GO:0016818	hydrolase activity, acting on acid anhydrides, in phosphorus-containing anhydrides	0.042332	13
GO:0005856	cytoskeleton	0.044912	20

Plant-specific GO terms significantly enriched for unigenes showing higher transcription in phase 2 ($P<0.05$)

GO term ID	Name	adjusted P value	Unigene number
GO:0044260	cellular macromolecule metabolic process	4.85E-11	224
GO:0043229	intracellular organelle	4.85E-11	632
GO:0043231	intracellular membrane-bounded organelle	4.85E-11	609
GO:0009536	plastid	4.85E-11	298
GO:0043227	membrane-bounded organelle	4.85E-11	609
GO:0043170	macromolecule metabolic process	4.85E-11	267
GO:0043226	organelle	4.85E-11	632
GO:0044444	cytoplasmic part	4.85E-11	548
GO:0005739	mitochondrion	5.76E-11	221
GO:0005515	protein binding	5.76E-11	97

GO:0003676	nucleic acid binding	1.79E-10	142
GO:0005622	intracellular	1.86E-09	24
GO:0005215	transporter activity	2.91E-09	142
GO:0016020	membrane	2.91E-09	482
GO:0005737	cytoplasm	2.91E-09	516
GO:0019538	protein metabolic process	2.59E-08	145
GO:0034641	cellular nitrogen compound metabolic process	1.34E-07	170
GO:0006139	nucleobase, nucleoside, nucleotide and nucleic acid metabolic process	1.34E-07	170
GO:0006807	nitrogen compound metabolic process	1.34E-07	170
GO:0090304	nucleic acid metabolic process	2.38E-07	138
GO:0005634	nucleus	6.20E-07	80
GO:0006259	DNA metabolic process	7.11E-07	40

GO:0003723	RNA binding	1.06E-06	19
GO:0051234	establishment of localization	1.51E-06	156
GO:0006810	transport	1.51E-06	156
GO:0043412	macromolecule modification	1.90E-06	72
GO:0006464	protein modification process	1.90E-06	72
GO:0044267	cellular protein metabolic process	1.90E-06	90
GO:0065007	biological regulation	4.90E-05	32
GO:0009987	cellular process	1.03E-04	542
GO:0050789	regulation of biological process	1.20E-04	28
GO:0044424	intracellular part	1.20E-04	1078
GO:0007049	cell cycle	1.29E-04	3
GO:0023046	signaling process	1.29E-04	28

GO:0007165	signal transduction	1.29E-04	28
GO:0023060	signal transmission	1.29E-04	28
GO:0050794	regulation of cellular process	1.29E-04	28
GO:0044237	cellular metabolic process	2.38E-04	362
GO:0005488	binding	2.82E-04	665
GO:0016740	transferase activity	3.07E-04	231
GO:0003677	DNA binding	3.26E-04	121
GO:0003682	chromatin binding	6.15E-04	5
GO:0016043	cellular component organization	8.00E-04	22
GO:0060089	molecular transducer activity	0.001292	18
GO:0004871	signal transducer activity	0.001292	18
GO:0044238	primary metabolic process	0.002297	489

GO:0034645	cellular macromolecule biosynthetic process	0.002577	113
GO:0009059	macromolecule biosynthetic process	0.002577	113
GO:0044249	cellular biosynthetic process	0.002577	113
GO:0016301	kinase activity	0.002652	104
GO:0016772	transferase activity, transferring phosphorus-containing groups	0.002652	104
GO:0006350	transcription	0.003068	53
GO:0017111	nucleoside-triphosphatase activity	0.004415	1
GO:0016462	pyrophosphatase activity	0.004415	1
GO:0016817	hydrolase activity, acting on acid anhydrides	0.004415	1
GO:0003774	motor activity	0.004415	1
GO:0016818	hydrolase activity, acting on acid anhydrides, in phosphorus-containing anhydrides	0.004415	1
GO:0005794	Golgi apparatus	0.006617	3

GO:0006091	generation of precursor metabolites and energy	0.010045	5
GO:0006950	response to stress	0.011691	73
GO:0005975	carbohydrate metabolic process	0.011691	87
GO:0003824	catalytic activity	0.011691	690
GO:0005777	peroxisome	0.011691	16
GO:0042579	microbody	0.011691	16
GO:0006629	lipid metabolic process	0.011691	68
GO:0019748	secondary metabolic process	0.014342	32
GO:0000166	nucleotide binding	0.017742	214
GO:0005623	cell	0.018543	176
GO:0005576	extracellular region	0.021554	34
GO:0016265	death	0.021554	34

GO:0008219	cell death	0.021554	34
GO:0030529	ribonucleoprotein complex	0.021554	34
GO:0032991	macromolecular complex	0.021554	34
GO:0005840	ribosome	0.021554	34
GO:0031975	envelope	0.025861	18
GO:0031967	organelle envelope	0.025861	18
GO:0005635	nuclear envelope	0.025861	18
GO:0005618	cell wall	0.037573	7
GO:0030312	external encapsulating structure	0.04165	5
GO:0008135	translation factor activity, nucleic acid binding	0.046677	2
