# Supplementary material for: De Novo Sequencing and Characterization of the Floral Transcriptome of Dendrocalamus latiflorus (Poaceae: Bambusoideae)
Source: PLoS One. 2012 Aug 14;7(8):e42082. doi: 10.1371/journal.pone.0042082 (PMC3419236; doi:10.1371/journal.pone.0042082)
Supplement: Table S4 — Enriched KEGG pathways for unigenes showing differential transcript abundance. A list of KEGG pathways enriched for unigenes showing higher transcript abundance in phase 1 and phase 2, respectively (p<0.05). (PDF) [file pone.0042082.s008.pdf]

KEGG pathways significantly enriched for unigenes showing higher transcription in phase 1 ($P<0.05$)

Pathway names	ko names	adjusted P value	Unigene number
Cell cycle	ko04110	0.00E+00	166
DNA replication	ko03030	0.00E+00	112
DNA replication proteins	ko03032	0.00E+00	358
DNA repair and recombination proteins	ko03400	3.69E-14	327
Cytoskeleton proteins	ko04812	5.87E-10	101
Chromosome	ko03036	4.31E-09	495
Mismatch repair	ko03430	4.40E-07	49
Base excision repair	ko03410	8.54E-06	63
Progesterone-mediated oocyte maturation	ko04914	1.36E-05	54
Homologous recombination	ko03440	1.32E-04	43
Pyrimidine metabolism	ko00240	1.92E-04	156
Nucleotide excision repair	ko03420	4.33E-04	64
Primary immunodeficiency	ko05340	7.53E-03	4
p53 signaling pathway	ko04115	1.38E-02	34
Purine metabolism	ko00230	2.46E-02	159
Transcription factors	ko03000	2.74E-02	140

KEGG pathways significantly enriched for unigenes showing higher transcription in phase 2 (P<0.05)

Pathway names	Ko names	adjusted P value	Unigene number
gamma-Hexachlorocyclohexane degradation	ko00361	0.00E+00	118
Limonene and pinene degradation	ko00903	0.00E+00	116
Naphthalene and anthracene degradation	ko00626	0.00E+00	105
Phenylpropanoid biosynthesis	ko00940	0.00E+00	140
Starch and sucrose metabolism	ko00500	0.00E+00	211
Stilbenoid, diarylheptanoid and gingerol biosynthesis	ko00945	0.00E+00	110
Flavonoid biosynthesis	ko00941	2.66E-15	54
Galactose metabolism	ko00052	2.66E-15	81
Nitrogen metabolism	ko00910	2.89E-13	64
Cytochrome P450	ko00199	1.22E-12	56
Pentose and glucuronate interconversions	ko00040	1.60E-12	64
Cyanoamino acid metabolism	ko00460	1.26E-11	67
Isoquinoline alkaloid biosynthesis	ko00950	2.09E-11	30
Linoleic acid metabolism	ko00591	5.14E-11	40
alpha-Linolenic acid metabolism	ko00592	6.89E-11	56
Tyrosine metabolism	ko00350	6.89E-11	56
Tryptophan metabolism	ko00380	4.00E-10	63
Phenylalanine metabolism	ko00360	2.04E-09	71
Styrene degradation	ko00643	2.41E-07	15
D-Glutamine and D-glutamate metabolism	ko00471	5.72E-07	7
Metabolism of xenobiotics by cytochrome P450	ko00980	1.79E-06	40
Drug metabolism - cytochrome P450	ko00982	4.28E-06	42
Indole alkaloid biosynthesis	ko00901	5.29E-06	18
Brassinosteroid biosynthesis	ko00905	1.13E-05	11
3-Chloroacrylic acid degradation	ko00641	1.80E-05	24
Anthocyanin biosynthesis	ko00942	2.45E-05	10
Retinol metabolism	ko00830	3.03E-05	25
beta-Alanine metabolism	ko00410	6.01E-05	33
Valine, leucine and isoleucine degradation	ko00280	6.01E-05	38
Fatty acid metabolism	ko00071	7.99E-05	46
Steroid biosynthesis	ko00100	8.98E-05	34
Alanine, aspartate and glutamate metabolism	ko00250	1.13E-04	46
Tropane, piperidine and pyridine alkaloid biosynthesis	ko00960	1.35E-04	15

Olfactory transduction	ko04740	1.40E-04	28
Biosynthesis of unsaturated fatty acids	ko01040	2.10E-04	27
ABC transporters	ko02010	3.12E-04	55
Taurine and hypotaurine metabolism	ko00430	3.12E-04	10
Transporters	ko02000	4.67E-04	66
Glycolysis / Gluconeogenesis	ko00010	5.46E-04	84
Sphingolipid metabolism	ko00600	6.34E-04	34
Methane metabolism	ko00680	6.44E-04	44
Nicotinate and nicotinamide metabolism	ko00760	7.37E-04	16
Neurotrophin signaling pathway	ko04722	8.68E-04	74
Diterpenoid biosynthesis	ko00904	9.31E-04	17
1,2-Dichloroethane degradation	ko00631	1.37E-03	10
Zeatin biosynthesis	ko00908	1.43E-03	12
Betalain biosynthesis	ko00965	2.02E-03	7
Flavone and flavonol biosynthesis	ko00944	2.02E-03	13
Terpenoid backbone biosynthesis	ko00900	2.46E-03	32
GnRH signaling pathway	ko04912	5.96E-03	52
Carotenoid biosynthesis	ko00906	6.85E-03	25
Glyoxylate and dicarboxylate metabolism	ko00630	8.73E-03	27
Arginine and proline metabolism	ko00330	1.24E-02	38
1- and 2-Methylnaphthalene degradation	ko00624	1.62E-02	15
Glycosphingolipid biosynthesis - globoseries	ko00603	1.62E-02	13
PPAR signaling pathway	ko03320	1.66E-02	24
Apoptosis	ko04210	1.70E-02	39
Oxidative phosphorylation	ko00190	1.76E-02	69
Cellular antigens	ko04090	1.90E-02	33
C21-Steroid hormone metabolism	ko00140	2.20E-02	2
Carbon fixation in photosynthetic organisms	ko00710	2.20E-02	48
Monoterpenoid biosynthesis	ko00902	2.20E-02	2
Transcription factors	ko03000	2.20E-02	136
Glucosinolate biosynthesis	ko00966	2.71E-02	8
Other glycan degradation	ko00511	2.83E-02	23
Glycosphingolipid biosynthesis - lacto and neolacto series	ko00601	2.91E-02	5
Lipid biosynthesis proteins	ko01004	3.08E-02	41
Glycosaminoglycan degradation	ko00531	3.45E-02	10
Glycerophospholipid metabolism	ko00564	3.53E-02	53
Ether lipid metabolism	ko00565	3.75E-02	31

Propanoate metabolism	ko00640	4.03E-02	27
Benzoate degradation via hydroxylation	ko00362	4.29E-02	2
