# Supplementary material for: De Novo Sequencing and Characterization of the Floral Transcriptome of Dendrocalamus latiflorus (Poaceae: Bambusoideae)
Source: PLoS One. 2012 Aug 14;7(8):e42082. doi: 10.1371/journal.pone.0042082 (PMC3419236; doi:10.1371/journal.pone.0042082)
Supplement: Table S5 — Representatives of putative flowering-time genes in D.latiflorus . A list of the D. latiflorus putative flowering time control genes and their possible functions. (PDF) [file pone.0042082.s009.pdf]

Table 2 Representatives of putative flowering-time genes in *Dendrocalamus latiflorus*

Representative unigene ID	Unigene Length	Accession number (Protein)	Identity	Align length (Amino acid)	E-value	Protein function (species)	Transcription factor family
**Photoperiod pathway**							
Unigene71172_All	884	AAL99264	0.79	177	6.00E-66	CO5 [*Hordeum vulgare* subsp. *vulgare*]	C2C2-CO-like
Unigene6072_All	806	AAL99264	0.82	147	6.00E-58	CO5 [*Hordeum vulgare* subsp. *vulgare*]	C2C2-CO-like
Unigene112514_All	1078	NP_001148229	0.76	134	6.00E-98	CO6 [*Zea mays*]	C2C2-CO-like
Unigene116927_All	232	NP_001148229	0.75	81	8.00E-25	CO6 [*Zea mays*]	C2C2-CO-like
Unigene124635_All	436	NP_001148229	0.85	70	1.00E-32	CO6 [*Zea mays*]	C2C2-CO-like
Unigene49735_All	564	AAL99269	0.66	42	3.00E-07	CO7 [*Hordeum vulgare* subsp. *vulgare*]	
Unigene17806_All	419	AAL99270	0.71	140	5.00E-42	CO8 [*Hordeum vulgare* subsp. *vulgare*]	C2C2-CO-like
Unigene142123_All	266	NP_001148275	0.76	94	2.00E-29	CO8 [*Zea mays*]	C2C2-CO-like
Unigene34101_All	620	ABL11477	0.73	169	5.00E-63	ELF3 [*Triticum aestivum*]	
Unigene35958_All	248	ABL11477	0.68	79	2.00E-23	ELF3 [*Triticum aestivum*]	
Unigene36395_All	376	ABL11477	0.64	129	3.00E-33	ELF3 [*Triticum aestivum*]	
Unigene40975_All	1128	ABL11477	0.64	211	2.00E-84	ELF3 [*Triticum aestivum*]	
Unigene1217_All	825	ABL1147	0.66	283	2.00E-90	ELF3 [*Triticum aestivum*]	
Unigene86406_All	261	ABL11477	0.96	30	6.00E-08	ELF3 [*Triticum aestivum*]	
Unigene63386_All	384	ABA94824	0.8	46	2.00E-12	ELF4 [*Oryza sativa* subsp. *japonica*]	
Unigene123448_All	355	ABA94824	0.95	73	1.00E-33	ELF4 [*Oryza sativa* subsp. *japonica*]	
Unigene123585_All	326	ABA94824	0.93	33	4.00E-10	ELF4 [*Oryza sativa* subsp. *japonica*]	
Unigene31669_All	404	NP_001148036	0.83	55	1.00E-15	ELF4 [*Zea mays*]	
Unigene23923_All	325	NP_001148036	0.87	55	1.00E-16	ELF4 [*Zea mays*]	
Unigene158_All	966	BAA94422	0.93	321	1.00E-17	COP1 [*Oryza sativa* subsp. *japonica*]	
Unigene137654_All	215	BAD87451	1	71	6.00E-36	Cop1 [*Oryza sativa* subsp. *japonica*]	Orphans
Unigene90233_All	205	ACG26686	0.8	68	2.00E-25	LHY [*Zea mays*]	

Unigene142505_All	272	ACG27662	0.78	91	3.00E-32	LHY [*Zea mays*]	
Unigene53198_All	707	BAD37964	0.9	144	3.00E-83	ID1 [*Oryza sativa* subsp. *japonica*]	C2H2
Unigene6817_All	954	BAD72204	0.66	305	2.00E-87	ID1 [*Oryza sativa* subsp. *japonica*]	C2H2
Unigene10252_All	245	BAD72204	0.65	41	8.00E-09	ID1 [*Oryza sativa* subsp. *japonica*]	
Unigene111896_All	382	BAD17526	0.72	126	2.00E-39	ID1 [*Oryza sativa* subsp. *japonica*]	C2H2
Unigene108149_All	480	ABC58220	0.64	133	2.00E-53	ID1 [*Lolium perenne*]	C2H2
Unigene18709_All	335	ABC58220	0.69	100	3.00E-30	ID1 [*Lolium perenne*]	C2H2
Unigene110772_All	571	AAV51392	0.91	56	1.00E-20	INDETERMINATE-related protein 9 [*Zea mays*]	
Unigene67635_All	211	NP_001151536	0.92	41	8.00E-14	PRR95 [*Zea mays*]	
Unigene19901_All	227	NP_001151536	0.63	76	2.00E-19	PRR95 [*Zea mays*]	Orphans
Unigene20124_All	231	NP_001151536	0.64	76	3.00E-22	PRR95 [*Zea mays*]	Orphans
**Autonomous pathway**							
Unigene72613_All	606	NP_001148070	0.84	201	4.00E-94	FLD [*Zea mays*]	SWI/SNF-SWI3
Unigene82146_All	843	Q01H90	0.87	208	1.00E-130	FLD [*Oryza sativa* subsp. *indica*]	SWI/SNF-SWI3
Unigene138536_All	222	AAT72461	0.83	73	2.00E-30	FY [*Lolium perenne*]	
Unigene105111_All	1175	EEE56009	0.94	283	1.00E-161	FY [*Arabidopsis thaliana*]	
Unigene137679_All	215	AAT72460	0.87	71	2.00E-28	FCA [*Lolium perenne*]	
Unigene3202_All	615	AAQ74971	0.82	50	2.00E-16	OsFCA3 [*Oryza sativa* subsp. *indica*]	
Unigene88285_All	579	XP_001692436	0.57	42	3E-06	DCL [*Chlamydomonas reinhardtii*]	
Unigene55543_All	215	XP_001692436	0.48	47	8E-06	DCL [*Chlamydomonas reinhardtii*]	
Unigene79914_All	1283	Q10HL3	0.75	361	1E-180	DCL2A [*Oryza sativa* subsp. *japonica*]	
Unigene146116_All	501	Q10HL3	0.8	166	8E-71	DCL2A [*Oryza sativa* subsp. *japonica*]	
Unigene102026_All	592	Q10HL3	0.71	112	1E-78	DCL2A [*Oryza sativa* subsp. *japonica*]	
Unigene118340_All	2757	Q10HL3	0.73	643	0.0	DCL2A [*Oryza sativa* subsp. *japonica*]	
Unigene143107_All	283	Q5N870	0.91	94	8E-46	DCL3A [*Oryza sativa* subsp. *japonica*]	
Unigene80238_All	484	Q5N870	0.55	94	2E-44	DCL3A [*Oryza sativa* subsp. *japonica*]	

Unigene26771_All	535	Q5N870	0.86	177	9E-87	DCL3A [*Oryza sativa* subsp. *japonica*]	
Unigene51749_All	250	Q5N870	0.91	82	5E-39	DCL3A [*Oryza sativa* subsp. *japonica*]	
**Vernalization pathway**							
Unigene145322_All	366	ABF97823	0.98	121	1.00E-65	MSI1 [*Oryza sativa* subsp. *japonica*]	
**GA-signaling pathway**							
Unigene126197_All	323	ACN80068	0.92	105	2.00E-46	d8 [*Zea luxurians*]	GRAS
Unigene24443_All	346	ACN80068	1	115	1.00E-63	d8 [*Zea luxurians*]	GRAS
Unigene67403_All	2122	CAA67000	0.47	400	1.00E-146	GAMyb [*Oryza sativa* subsp. *indica*]	MYB
Unigene114898_All	1403	CAA67000	0.49	353	1.00E-80	GAMyb [*Oryza sativa* subsp. *indica*]	MYB
Unigene41432_All	460	A2WW87	0.81	116	3.00E-50	GAMYB [*Oryza sativa* subsp. *indica*]	MYB
Unigene132859_All	578	A2WW87	0.81	74	6.00E-60	GAMYB [*Oryza sativa* subsp. *indica*]	MYB
Unigene37234_All	1099	BAE92565	0.94	244	1.00E-135	GAMYB [*Triticum monococcum*]	MYB
Unigene5334_All	1589	CAA61021	0.87	350	0	GAMyb [*Hordeum vulgare* subsp. *vulgare*]	MYB
Unigene46215_All	263	AAT40121	0.94	87	1.00E-39	GAMYB-binding [*Hordeum vulgare* subsp. *vulgare*]	TRAF
Unigene66567_All	541	CAJ75637	0.71	59	1.00E-33	GAMYB [*Brachypodium sylvaticum*]	
Unigene142610_All	274	CAJ75637	0.85	80	1.00E-37	GAMYB [*Brachypodium sylvaticum*]	
Unigene6495_All	643	CAJ75637	0.88	113	3.00E-54	GAMYB [*Brachypodium sylvaticum*]	
Unigene10713_All	537	CAJ75637	0.86	113	7.00E-53	GAMYB [*Brachypodium sylvaticum*]	
Unigene32417_All	1513	BAE46986	0.43	326	9.00E-75	GAMYBL1 [*Oryza sativa* subsp. *japonica*]	MYB
Unigene67869_All	299	ABM55475	0.77	57	4.00E-19	GAox [*Oryza sativa* subsp. *japonica*]	
Unigene84529_All	321	ABY49031	0.9	54	4.00E-35	GA3ox [*Rhynchoryza subulata*]	
Unigene31211_All	423	ACU40946	0.91	93	3.00E-58	GA20OX [*Dasypyrum villosum*]	
Unigene35600_All	622	ACU40941	0.82	63	4.00E-38	GA20OX [*Dasypyrum villosum*]	
Unigene139544_All	232	NP_001149522	0.8	77	1.00E-29	GA20OX2 [*Zea mays*]	
Unigene41987_All	589	NP_001146872	0.85	48	3.00E-26	gibberellin 2-beta-dioxygenase [*Zea mays*]	
Unigene48392_All	340	CAP64326	0.89	113	4.00E-56	GID1 [*Saccharum officinarum*]	NAC

Unigene66422_All	771	CAO98733	0.81	107	3.00E-46	GID1L [*Hordeum vulgare* subsp. *vulgare*]	
Unigene115709_All	697	CAO98733	0.81	107	7.00E-45	GID1L [*Hordeum vulgare* subsp. *vulgare*]	
Unigene82828_All	404	NP_001150146	0.72	136	2.00E-46	GID1L2 [*Zea mays*]	TIG
Unigene125430_All	309	NP_001150146	0.78	100	5.00E-37	GID1L2 [*Zea mays*]	TIG
Unigene133170_All	391	NP_001150234	0.74	75	1.00E-25	GID1L2 [*Zea mays*]	
Unigene39030_All	404	NP_001150584	0.96	28	6.00E-09	GID1L2 [*Zea mays*]	
Unigene84542_All	303	NP_001150584	0.87	87	1.00E-40	GID1L2 [*Zea mays*]	
Unigene108784_All	268	NP_001151089	0.67	92	5.00E-24	GID1L2 [*Zea mays*]	
Unigene55148_All	1038	ACG37635	0.6	145	3.00E-80	GID1L2 [*Zea mays*]	
Unigene20303_All	290	NP_001152731	0.95	21	9.00E-07	gibberellin-regulated protein 1 [*Zea mays*]	
**Floral meristem identity genes**							
Unigene90859_All	208	ACG28758	0.97	69	1.00E-29	AP1 [*Zea mays*]	
Unigene102981_All	286	ACG28758	1	75	1.00E-36	AP1 [*Zea mays*]	
Unigene1139_All	656	Q10CQ1	0.86	138	2.00E-58	OsMADS14 [*Oryza sativa* subsp. *japonica*]	MADS
Unigene26434_All	484	Q2V0P1	0.8	162	5.00E-70	MADS58 [*Oryza sativa* subsp. *japonica*]	MADS
**Other flowering genes**							
Unigene62748_All	262	BAF64440	0.73	87	1.00E-28	HAP2 [*Oryza sativa* subsp. *japonica*]	CCAAT
Unigene76546_All	984	BAF64440	0.68	303	1.00E-100	HAP2 [*Oryza sativa* subsp. *japonica*]	CCAAT
Unigene108326_All	1690	BAF64440	0.52	70	3.00E-08	HAP2 [*Oryza sativa* subsp. *japonica*]	
Unigene26823_All	545	BAF64435	0.76	155	5.00E-60	HAP2 [*Oryza sativa* subsp. *japonica*]	CCAAT
Unigene7704_All	770	BAF64441	0.78	177	4.00E-73	HAP2 [*Oryza sativa* subsp. *japonica*]	CCAAT
Unigene13215_All	945	BAF64441	0.76	192	1.00E-83	HAP2 [*Oryza sativa* subsp. *japonica*]	CCAAT
Unigene106893_All	321	BAF64445	0.7	51	6.00E-12	HAP3 [*Oryza sativa* subsp. *japonica*]	CCAAT
Unigene27847_All	580	ABF96565	0.68	98	1.00E-28	WHAP3 [*Oryza sativa* subsp. *japonica*]	CCAAT
Unigene30887_All	437	ABF96565	0.73	140	8.00E-54	WHAP3 [*Oryza sativa* subsp. *japonica*]	CCAAT

Unigene145865_All	435	NP_001106241	0.95	118	6.00E-62	ZCN2 [*Zea mays*]	
Unigene34590_All	486	NP_001106241	0.8	51	1.00E-14	ZCN2 [*Zea mays*]	
Unigene105152_All	657	NP_001106253	0.93	61	2.00E-28	ZCN16 [*Zea mays*]	
Unigene35282_All	660	NP_001151095	0.9	116	4.00E-57	ARP6 [*Zea mays*]	
Unigene29453_All	380	A2XX39	0.97	80	1.00E-56	RFL [*Oryza sativa* subsp. *indica*]	LFY
Unigene79416_All	331	AAY33602	0.82	111	2.00E-45	RFL [*Oryza meridionalis*]	LFY
Unigene14137_All	560	ABD15908	0.57	187	8.00E-50	EMF1 [*Oryza longistaminata*]	
Unigene117429_All	452	ABD15906	0.55	149	5.00E-38	EMF1 [*Oryza meridionalis*]	
Unigene80515_All	723	BAD81323	0.49	201	2.00E-35	EMF1 [*Oryza sativa* subsp. *japonica*]	
Unigene80559_All	239	BAD81323	0.62	29	4.00E-06	EMF1 [*Oryza sativa* subsp. *japonica*]	
Unigene105105_All	1164	BAD81323	0.41	172	3.00E-46	EMF1 [*Oryza sativa* subsp. *japonica*]	
Unigene115434_All	243	BAD81323	0.47	85	2.00E-09	EMF1 [*Oryza sativa* subsp. *japonica*]	
Unigene26696_All	523	ABB92268	0.96	162	2.00E-92	MSI [*Triticum aestivum*]	
Unigene81301_All	226	ABM81546	0.96	27	4.00E-08	VIN3 [*Triticum aestivum*]	
Unigene103609_All	325	ABM81546	0.79	108	1.00E-42	VIN3 [*Triticum aestivum*]	
Unigene5211_All	536	ABM81546	0.72	141	7.00E-52	VIN3 [*Triticum aestivum*]	
Unigene9113_All	432	ABM81546	0.74	144	3.00E-52	VIN3 [*Triticum aestivum*	
Unigene29774_All	679	ABM81546	0.81	225	1.00E-102	VIN3 [*Triticum aestivum*]	
Unigene27336_All	3034	NP_001065771	0.71	121	7.00E-38	FDR2 [*Oryza sativa*]	
Unigene32114_All	732	BAD45362	0.9	109	6.00E-51	TFL1 [*Oryza sativa* subsp. *japonica*]	
Unigene19766_All	563	BAD45362	0.87	33	1.00E-09	TFL1 [*Oryza sativa* subsp. *japonica*]	
Unigene3_All	523	AAR87240	0.75	44	1.00E-07	AP1 [*Oryza sativa* subsp. *japonica*]	
Unigene2653_All	918	AAR87240	0.61	103	3.00E-20	AP1 [*Oryza sativa* subsp. *japonica*]	MADS
Unigene80271_All	265	AAW78375	0.76	88	2.00E-29	AP2D10 [*Oryza sativa* subsp. *japonica*]	AP2-EREBP
Unigene145190_All	357	AAW78375	0.78	119	4.00E-50	AP2D10 [*Oryza sativa* subsp. *japonica*]	AP2-EREBP
Unigene36290_All	398	ABR25960	0.61	137	2.00E-32	AP2d23 [*Oryza sativa* subsp. *indica*]	AP2-EREBP

Unigene126469_All	372	ABR25960	0.69	136	3.00E-41	AP2d23 [*Oryza sativa* subsp. *indica*]	AP2-EREBP
Unigene41092_All	520	ABF95597	0.79	119	4.00E-71	AP2 [*Oryza sativa* subsp. *japonica*]	AP2-EREBP
Unigene86676_All	316	ABF99086	0.74	82	1.00E-27	AP2 [*Oryza sativa* subsp. *japonica*]	AP2-EREBP
Unigene56516_All	904	BAD17329	0.64	147	4.00E-71	AP2 [*Oryza sativa* subsp. *japonica*]	AP2-EREBP
Unigene9037_All	708	BAD25897	0.67	237	8.00E-71	AP2 [*Oryza sativa* subsp. *japonica*]	AP2-EREBP
Unigene52813_All	386	AAP83321	0.88	79	2.00E-36	AP2 [*Oryza sativa* subsp. *japonica*]	AP2-EREBP
Unigene59694_All	235	BAH57731	0.69	46	8.00E-12	AP2 [*Triticum aestivum*]	AP2-EREBP
Unigene70504_All	404	BAH57730	0.71	76	4.00E-32	AP2 [*Triticum aestivum*]	AP2-EREBP
Unigene79815_All	314	BAH57731	0.72	68	8.00E-19	AP2 [*Triticum aestivum*]	AP2-EREBP
Unigene83706_All	397	BAH57731	0.73	68	6.00E-19	AP2 [*Triticum aestivum*]	AP2-EREBP
Unigene52918_All	349	ACU44657	0.69	113	2.00E-29	AP2 [*Oryza sativa* subsp. *indica*]	AP2-EREBP
Unigene54745_All	298	ACU44657	0.65	93	3.00E-20	AP2 [*Oryza sativa* subsp. *indica*]	AP2-EREBP
Unigene102234_All	345	ACU44657	0.73	86	9.00E-26	AP2 [*Oryza sativa* subsp. *indica*]	AP2-EREBP
Unigene30435_All	535	ACU44657	0.69	71	4.00E-18	AP2 [*Oryza sativa* subsp. *indica*]	AP2-EREBP
Unigene2621_All	766	ABC71547	0.65	127	5.00E-47	AP2 [*Coix lacryma-jobi*]	AP2-EREBP
Unigene37432_All	587	ABC71546	0.79	127	2.00E-50	AP2 [*Setaria italica*]	AP2-EREBP
Unigene79153_All	351	ABC71546	0.76	81	3.00E-30	AP2 [*Setaria italica*]	AP2-EREBP
Unigene43201_All	462	ABY55158	0.98	64	4.00E-29	AP2/EREBP [*Oryza sativa* subsp. *indica*]	AP2-EREBP
Unigene102677_All	287	ABY55158	0.91	95	3.00E-44	AP2/EREBP [*Oryza sativa* subsp. *indica*]	AP2-EREBP
Unigene145668_All	405	ACJ74070	0.84	38	1.00E-11	AP2/ERF [*Oryza sativa* subsp. *japonica*]	AP2-EREBP
Unigene93714_All	229	AAP56252	0.75	62	2.00E-20	DBF1 [*Oryza sativa* subsp. *japonica*]	AP2-EREBP
Unigene120684_All	283	ABD72476	0.77	40	6.00E-16	WRI1 [*Brassica napus*]	AP2-EREBP
Unigene136636_All	208	AAO38209	0.77	68	3.00E-25	DRF1 [*Hordeum vulgare*]	AP2-EREBP
Unigene46736_All	605	ABG90940	0.86	102	2.00E-65	AP3 [*Joinvillea ascendens*]	MADS
Unigene31051_All	537	AAS48127	0.8	57	4.00E-38	PI [*Hordeum vulgare* subsp. *vulgare*]	MADS
Unigene61519_All	319	AAR32118	0.51	80	4.00E-12	MADS [*Dendrocalamus latiflorus*]	MADS

Unigene122040_All	246	AAR32119	0.88	84	4.00E-35	MADS [*Dendrocalamus latiflorus*]	MADS
Unigene50496_All	851	BAF75016	0.87	184	3.00E-90	MADS [*Triticum aestivum*]	MADS
Unigene23470_All	510	CAE53900	0.78	102	3.00E-39	MADS [*Triticum aestivum*]	MADS
Unigene132755_All	617	BAD10102	0.73	78	8.00E-53	MADS [*Oryza sativa* subsp. *japonica*]	MADS
Unigene72676_All	417	BAD45640	0.75	40	1.00E-14	MADS [*Oryza sativa* subsp. *japonica*]	MADS
Unigene134101_All	494	BAD45640	0.6	170	4.00E-46	MADS [*Oryza sativa* subsp. *japonica*]	MADS
Unigene106009_All	313	NP_001105152	0.61	97	5.00E-23	MADS1 [*Zea mays*]	MADS
Unigene107374_All	422	NP_001105152	0.91	78	7.00E-34	MADS1 [*Zea mays*]	MADS
Unigene44535_All	478	ADA72020	0.86	51	5.00E-18	MADS1 [*Fargesia nitida*]	MADS
Unigene93522_All	227	AAT37484	0.88	34	8.00E-11	MADS5 [*Dendrocalamus latiflorus*]	MADS
Unigene105754_All	406	AAT37484	0.97	39	5.00E-15	MADS5 [*Dendrocalamus latiflorus*]	MADS
Unigene24549_All	348	AAT37488	0.99	116	5.00E-59	MADS9 [*Dendrocalamus latiflorus*]	MADS
Unigene40100_All	627	AAT37479	0.97	47	3.00E-21	MADS16 [*Dendrocalamus latiflorus*]	MADS
Unigene141320_All	254	AAT37480	1	84	3.00E-42	MADS17 [*Dendrocalamus latiflorus*]	MADS
Unigene43324_All	1381	ACX35552	0.77	253	4.00E-99	MADS17 [*Oryza sativa* subsp. *japonica*]	MADS
Unigene65412_All	640	Q0J8G8	0.97	116	5.00E-58	OsMADS26 [*Oryza sativa* subsp. *japonica*]	MADS
Unigene108994_All	507	Q0J8G8	0.91	161	8.00E-76	OsMADS26 [*Oryza sativa* subsp. *japonica*]	MADS
Unigene24617_All	1569	AAO47706	0.81	53	2.00E-44	MADS27 [*Oryza sativa* subsp. *japonica*]	MADS
Unigene3264_All	894	AAO47709	0.92	196	2.00E-99	MADS32 [*Oryza sativa* subsp. *japonica*]	MADS
Unigene80002_All	1099	ACG37013	0.78	47	3.00E-27	MADS34 [*Zea mays*]	MADS
Unigene132840_All	1072	ACG37013	0.69	102	1.00E-32	MADS34 [*Zea mays*]	MADS
Unigene114974_All	239	Q69TG5	0.81	43	5.00E-29	MADS55 [*Oryza sativa* subsp. *japonica*]	MADS
Unigene20467_All	948	BAD35842	0.84	171	4.00E-72	MADS55 [*Oryza sativa* subsp. *japonica*]	MADS
Unigene132147_All	542	AAO47712	0.48	155	4.00E-27	MADS57 [*Oryza sativa* subsp. *japonica*]	MADS
Unigene86443_All	350	AAS59823	0.91	96	9.00E-42	RMADS212 [*Oryza sativa*]	MADS
Unigene86226_All	310	AAS59830	0.82	34	6.00E-09	RMADS219 [*Oryza sativa*]	

Unigene81200_All	218	CAM59056	0.89	73	4.00E-30	WM12 [*Triticum aestivum*]	
Unigene69728_All	586	CAM59040	0.88	62	4.00E-38	WM1B [*Triticum aestivum*]	
Unigene28511_All	615	CAM59040	0.83	134	2.00E-53	WM1B [*Triticum aestivum*]	
Unigene13899_All	920	CAM59041	0.81	93	4.00E-34	WM2 [*Triticum aestivum*]	
Unigene78812_All	402	CAM59068	0.75	53	3.00E-15	WM22B [*Triticum aestivum*]	
Unigene133536_All	374	CAM59077	0.95	41	8.00E-22	WM29B [*Triticum aestivum*]	
Unigene37578_All	2658	CAM59082	0.76	56	2.00E-36	WM32A [*Triticum aestivum*]	MADS
Unigene6831_All	902	CAM59082	0.88	34	3.00E-25	WM32A [*Triticum aestivum*]	MADS
Unigene53328_All	251	CAM59043	0.67	34	1.00E-07	WM3B [*Triticum aestivum*]	
Unigene27372_All	759	ABF57914	0.66	186	2.00E-57	TaAGL1 [*Triticum aestivum*]	MADS
Unigene86176_All	473	ABF57951	0.64	108	1.00E-31	TaAGL12 [*Triticum aestivum*]	MADS
Unigene12527_All	886	ABF57951	0.67	120	2.00E-33	TaAGL12 [*Triticum aestivum*]	MADS
Unigene91358_All	211	ABF57933	0.95	70	2.00E-32	TaAGL31 [*Triticum aestivum*]	MADS
Unigene59274_All	564	ABF57941	0.41	85	1.00E-06	TaAGL41 [*Triticum aestivum*]	
Unigene115464_All	229	ABF57941	0.8	75	1.00E-26	TaAGL41 [*Triticum aestivum*]	MADS
Unigene109537_All	442	ABF57947	0.74	156	5.00E-50	TaAGL7 [*Triticum aestivum*]	MADS
Unigene50601_All	674	ABZ91910	0.53	222	3.00E-45	FDL6 [*Triticum aestivum*]	bZIP
Unigene134693_All	476	ABG80460	0.61	107	1.00E-23	FUL [*Lithachne humilis*]	MADS
Unigene80969_All	681	Q0JGI1	0.65	242	3.00E-69	SPL2 [*Oryza sativa* subsp. *japonica*]	SBP
Unigene122563_All	321	Q0JGI1	0.55	61	1.00E-09	SPL2 [*Oryza sativa* subsp. *japonica*]	SBP
Unigene13834_All	884	Q0JGI1	0.62	241	6.00E-64	SPL2 [*Oryza sativa* subsp. *japonica*]	SBP
Unigene38571_All	929	A2X0Q6	0.63	211	9.00E-61	SPL3 [*Oryza sativa* subsp. *indica*]	SBP
Unigene47199_All	902	A2X0Q6	0.65	191	3.00E-60	SPL3 [*Oryza sativa* subsp. *indica*]	SBP
Unigene69564_All	864	A2X0Q6	0.65	190	2.00E-58	SPL3 [*Oryza sativa* subsp. *indica*]	SBP
Unigene129373_All	242	A2X0Q6	0.71	82	3.00E-27	SPL3 [*Oryza sativa* subsp. *indica*]	SBP
Unigene36435_All	1404	Q75LH6	0.67	101	2.00E-36	SPL6 [*Oryza sativa* subsp. *japonica*]	SBP

Unigene123715_All	255	Q75LH6	0.84	51	4.00E-24	SPL6 [*Oryza sativa* subsp. *japonica*]	SBP
Unigene124634_All	304	Q75LH6	0.85	101	8.00E-46	SPL6 [*Oryza sativa* subsp. *japonica*]	SBP
Unigene8783_All	400	Q75LH6	0.75	132	6.00E-49	SPL6 [*Oryza sativa* subsp. *japonica*]	SBP
Unigene31770_All	543	Q01KM7	0.82	158	1.00E-75	SPL8 [*Oryza sativa* subsp. *indica*]	SBP
Unigene2551_All	717	Q01KM7	0.87	246	1.00E-119	SPL8 [*Oryza sativa* subsp. *indica*]	SBP
Unigene9005_All	276	Q01KM7	0.75	98	2.00E-32	SPL8 [*Oryza sativa* subsp. *indica*]	SBP
Unigene11540_All	555	Q01KM7	0.77	149	5.00E-63	SPL8 [*Oryza sativa* subsp. *indica*]	SBP
Unigene115408_All	204	Q6I576	0.81	64	3.00E-25	SPL9 [*Oryza sativa* subsp. *japonica*]	SBP
Unigene48546_All	446	A2YGR5	0.58	79	4.00E-13	SPL12 [*Oryza sativa* subsp. *indica*]	SBP
Unigene54782_All	221	A2YGR5	0.73	42	2.00E-11	SPL12 [*Oryza sativa* subsp. *indica*]	SBP
Unigene3233_All	371	A2YGR5	0.61	118	4.00E-30	SPL12 [*Oryza sativa* subsp. *indica*]	SBP
Unigene4410_All	283	A2YGR5	0.81	95	2.00E-34	SPL12 [*Oryza sativa* subsp. *indica*]	SBP
Unigene6423_All	273	A2YGR5	0.76	92	8.00E-32	SPL12 [*Oryza sativa* subsp. *indica*]	SBP
Unigene10531_All	341	A2YGR5	0.8	114	6.00E-44	SPL12 [*Oryza sativa* subsp. *indica*]	SBP
Unigene28253_All	909	A2YGR5	0.68	216	4.00E-74	SPL12 [*Oryza sativa* subsp. *indica*]	SBP
Unigene61689_All	275	Q6Z461	0.52	110	2.00E-18	SPL13 [*Oryza sativa* subsp. *japonica*]	SBP
Unigene65438_All	204	Q7EXZ2	0.63	69	5.00E-16	SPL14 [*Oryza sativa* subsp. *japonica*]	SBP
Unigene9070_All	620	Q6Z8M8	0.71	102	3.00E-39	SPL15 [*Oryza sativa* subsp. *japonica*]	SBP
Unigene107143_All	239	BAC79948	0.7	65	9.00E-24	CIP7 [*Oryza sativa* subsp. *japonica*]	
Unigene68803_All	605	BAC21482	0.75	201	2.00E-76	CIP7 [*Oryza sativa* subsp. *japonica*]	
Unigene103010_All	288	AAO19379	0.71	97	3.00E-28	CBF [*Oryza sativa* subsp. *japonica*]	CCAAT
Unigene3632_All	867	AAM89287	0.71	67	1.00E-26	SET118 [*Zea mays*]	
Unigene14074_All	323	AAQ94319	0.86	67	4.00E-28	MAPK6 [*Zea mays*]	
Unigene31362_All	388	ABB77210	0.97	128	3.00E-66	EMF2 [*Dendrocalamus latiflorus*]	
Unigene33614_All	779	ABB77210	0.75	76	5.00E-46	EMF2 [*Dendrocalamus latiflorus*]	
Unigene33821_All	669	ABB77210	0.92	198	1.00E-107	EMF2 [*Dendrocalamus latiflorus*]	

Unigene44490_All	215	ABB77210	0.91	35	6.00E-12	EMF2 [*Dendrocalamus latiflorus*]	
Unigene57795_All	1977	ABB77210	0.76	568	0	EMF2 [*Dendrocalamus latiflorus*]	
Unigene2822_All	476	ABB77210	0.91	158	2.00E-75	EMF2 [*Dendrocalamus latiflorus*]	
Unigene109531_All	2381	ABB77210	0.77	610	0	EMF2 [*Dendrocalamus latiflorus*]	
Unigene128517_All	910	ABB77210	1	43	3.00E-20	EMF2 [*Dendrocalamus latiflorus*]	
Unigene132620_All	261	ABB77210	1	28	3.00E-10	EMF2 [*Dendrocalamus latiflorus*]	
Unigene135222_All	200	ABB77210	1	66	5.00E-32	EMF2 [*Dendrocalamus latiflorus*]	
Unigene7144_All	629	ABB77210	0.46	121	5.00E-20	EMF2 [*Dendrocalamus latiflorus*]	
Unigene15928_All	444	ABB77210	0.94	35	9.00E-13	EMF2 [*Dendrocalamus latiflorus*]	
Unigene20623_All	241	ABB77210	0.97	39	2.00E-16	EMF2 [*Dendrocalamus latiflorus*]	
Unigene39436_All	996	BAD08170	0.83	181	9.00E-78	myb [*Oryza sativa* subsp. *japonica*]	G2-like
Unigene74701_All	463	BAD08170	0.78	52	3.00E-15	myb [*Oryza sativa* subsp. *japonica*]	G2-like
Unigene69295_All	415	BAC99778	0.85	142	6.00E-59	myb [*Oryza sativa* subsp. *japonica*]	G2-like
Unigene39749_All	371	ACG40365	0.84	59	3.00E-20	myb [*Zea mays*]	G2-like
Unigene132434_All	267	BAD04039	0.57	82	1.00E-16	Myb [*Oryza glaberrima*]	MYB
Unigene33935_All	391	ACF22740	0.71	35	1.00E-12	Myb [*Brachypodium distachyon*]	MYB
Unigene85953_All	310	ACF22740	0.56	110	1.00E-24	Myb [*Brachypodium distachyon*]	MYB
Unigene86125_All	294	ACF22740	0.61	95	4.00E-25	Myb [*Brachypodium distachyon*]	MYB
Unigene123027_All	362	ACF22740	0.57	83	2.00E-17	Myb [*Brachypodium distachyon*]	
Unigene14193_All	690	ACF22745	0.65	120	6.00E-42	Myb [*Brachypodium distachyon*]	MYB
Unigene48736_All	671	AAU43823	0.5	130	8.00E-26	myb [*Hordeum vulgare* subsp. *vulgare*]	MYB
Unigene78343_All	333	AAU43823	0.76	56	8.00E-14	myb [*Hordeum vulgare* subsp. *vulgare*]	MYB-related
Unigene97176_All	265	AAU43823	0.51	93	3.00E-16	myb [*Hordeum vulgare* subsp. *vulgare*]	MYB
Unigene131947_All	246	AAU43823	0.79	62	6.00E-20	myb [*Hordeum vulgare* subsp. *vulgare*]	MYB-related
Unigene142790_All	277	AAU43823	0.53	102	1.00E-20	myb [*Hordeum vulgare* subsp. *vulgare*]	MYB
Unigene145394_All	372	AAU43823	0.7	98	1.00E-22	myb [*Hordeum vulgare* subsp. *vulgare*]	MYB

Unigene80280_All	306	BAH03544	0.67	37	2.00E-11	Myb [*Triticum aestivum*]	MYB
Unigene82248_All	290	BAH03544	0.62	94	2.00E-22	Myb [*Triticum aestivum*]	MYB
Unigene98705_All	288	BAH03544	0.81	104	3.00E-42	Myb [*Triticum aestivum*]	MYB
Unigene113090_All	628	BAH03544	0.89	139	5.00E-64	Myb [*Triticum aestivum*]	MYB
Unigene138629_All	223	BAH03544	0.8	81	4.00E-30	Myb [*Triticum aestivum*]	MYB
Unigene90677_All	207	AAK08983	0.71	52	4.00E-15	JAMyb [*Oryza sativa* subsp. *japonica*]	MYB
Unigene99116_All	296	AAK08983	0.61	109	1.00E-27	JAMyb [*Oryza sativa* subsp. *japonica*]	MYB
Unigene26956_All	575	AAK08983	0.71	142	3.00E-51	JAMyb [*Oryza sativa* subsp. *japonica*]	MYB
Unigene83396_All	1013	ABC86569	0.63	178	7.00E-80	TaMYB1 [*Triticum aestivum*]	MYB
Unigene116944_All	1045	ABC86569	0.79	177	2.00E-73	TaMYB1 [*Triticum aestivum*]	MYB
Unigene5888_All	775	ABC86569	0.78	37	1.00E-07	TaMYB1 [*Triticum aestivum*]	
Unigene142562_All	273	ABC86569	0.77	83	3.00E-33	TaMYB1 [*Triticum aestivum*]	MYB
Unigene138939_All	226	BAD17383	0.92	75	5.00E-34	MYB [*Oryza sativa* subsp. *japonica*]	MYB-related
Unigene6382_All	843	BAD17383	0.67	280	1.00E-102	MYB [*Oryza sativa* subsp. *japonica*]	MYB-related
Unigene33568_All	566	ABQ01978	0.69	193	2.00E-67	MYBAl [*Aeluropus littoralis*]	MYB
Unigene4837_All	652	ABA96597	0.68	44	2.00E-13	MYB10 [*Oryza sativa* subsp. *japonica*]	MYB
Unigene93551_All	228	CAC85051	0.74	81	2.00E-22	Myb13 [*Oryza sativa*]	MYB
Unigene119190_All	279	CAC85051	0.72	102	1.00E-25	Myb13 [*Oryza sativa*]	MYB
Unigene7844_All	202	NP_001105086	0.87	66	5.00E-27	myb2 [*Zea mays*]	MYB
Unigene18567_All	204	NP_001105086	0.85	68	4.00E-26	myb2 [*Zea mays*]	MYB
Unigene120895_All	260	NP_001152064	0.72	91	4.00E-30	Myb4 [*Zea mays*]	MYB
Unigene83677_All	318	AAZ20440	0.82	81	2.00E-48	MYB24 [*Malus* x *domestica*]	MYB
Unigene21051_All	1608	ACN85217	0.8	339	1.00E-146	MYBCC [*Oryza punctata*]	G2-like
Unigene29196_All	1246	ACN85217	0.82	274	1.00E-126	MYBCC [*Oryza punctata*]	G2-like
Unigene29951_All	1001	NP_001152613	0.8	256	1.00E-113	MYBCC [*Zea mays*]	G2-like
Unigene50444_All	531	CAA50221	0.82	63	3.00E-22	MybHv5 [*Hordeum vulgare* subsp. *vulgare*]	MYB

Unigene87052_All	270	CAA50221	0.83	65	1.00E-24	MybHv5 [*Hordeum vulgare* subsp. *vulgare*]	MYB
Unigene88739_All	383	ABA96851	0.86	113	7.00E-55	Myb [*Oryza sativa* subsp. *japonica*]	MYB
Unigene24026_All	650	ABA96851	0.91	215	1.00E-117	Myb [*Oryza sativa* subsp. *japonica*]	MYB
Unigene92718_All	221	NP_001150001	0.84	73	1.00E-29	myb [*Zea mays*]	G2-like
Unigene115034_All	419	NP_001150001	0.7	94	4.00E-28	myb [*Zea mays*]	G2-like
Unigene106225_All	465	ABA99223	0.82	84	3.00E-33	Myb-related [*Oryza sativa* subsp. *japonica*]	MYB-related
Unigene17231_All	771	ABA99223	0.81	93	2.00E-36	Myb-related [*Oryza sativa* subsp. *japonica*]	MYB-related
Unigene42110_All	278	NP_001149973	0.68	92	6.00E-28	myb-related [*Zea mays*]	MYB-related
Unigene86396_All	417	BAF80451	0.96	33	7.00E-20	MYB-related [*Nicotiana tabacum*]	MYB
Unigene81181_All	217	P42863	1	32	3E-11	G6PIB [*Oryza sativa* subsp. *japonica*]	
Unigene137069_All	211	P49105	0.98	69	3E-32	G6PI [*Zea mays*]	
Unigene20126_All	231	P49105	0.88	75	3E-32	G6PI [*Zea mays*]	
Unigene22230_All	275	P49105	0.97	91	1E-45	G6PI [*Zea mays*]	
Unigene14854_All	495	BAD10250	0.85	164	4E-79	SKB1 [*Oryza sativa* subsp. *japonica*]	
Unigene76655_All	838	BAD10250	0.82	108	3E-42	SKB1 [*Oryza sativa* subsp. *japonica*]	
Unigene120966_All	892	BAD10250	0.83	133	6E-57	SKB1 [*Oryza sativa* subsp. *japonica*]	
